# Targeting mitochondrial dysfunction in amyotrophic lateral sclerosis: a systematic review and meta-analysis

**DOI:** 10.1093/braincomms/fcz009

**Published:** 2019-08-06

**Authors:** Arpan R Mehta, Rachel Walters, Fergal M Waldron, Suvankar Pal, Bhuvaneish T Selvaraj, Malcolm R Macleod, Giles E Hardingham, Siddharthan Chandran, Jenna M Gregory

**Affiliations:** 1 UK Dementia Research Institute, University of Edinburgh, Edinburgh, UK; 2 Centre for Clinical Brain Sciences, University of Edinburgh, Edinburgh, UK; 3 The Anne Rowling Regenerative Neurology Clinic, University of Edinburgh, Edinburgh, UK; 4 The Euan MacDonald Centre, University of Edinburgh, Edinburgh, UK; 5 Nuffield Department of Clinical Neurosciences, University of Oxford, Oxford, UK; 6 Institute of Evolutionary Biology and Centre for Immunity Infection and Evolution, University of Edinburgh, Ashworth Laboratories, Edinburgh, UK; 7 Centre for Discovery Brain Sciences, University of Edinburgh, Edinburgh, UK; 8 Centre for Brain Development and Repair, inStem, Bangalore, India; 9 MRC Centre for Regenerative Medicine, University of Edinburgh, Edinburgh, UK; 10 Edinburgh Neuroscience, University of Edinburgh, Edinburgh, UK; 11 MRC Edinburgh Brain Bank, Academic Department of Neuropathology, University of Edinburgh, Edinburgh, UK

**Keywords:** systematic review, meta-analysis, amyotrophic lateral sclerosis, mitochondria, modelling

## Abstract

Interventions targeting mitochondrial dysfunction have the potential to extend survival in preclinical models of amyotrophic lateral sclerosis. The aim of this systematic review was to assess the efficacy of targeting mitochondria as a potential therapeutic target in amyotrophic lateral sclerosis. Preclinical studies written in the English language were identified with no restrictions on publication date from PubMed, Medline and EMBASE databases. All studies adopting interventions targeting mitochondria to treat amyotrophic lateral sclerosis in genetic or drug-induced organism models were considered for inclusion. A total of 76 studies were included in the analysis. Survival data were extracted, and the meta-analysis was completed in RevMan 5 software. We show that targeting mitochondrial dysfunction in amyotrophic lateral sclerosis results in a statistically significant improvement in survival (*Z *=* *5.31; *P *<* *0.00001). The timing of administration of the intervention appears to affect the improvement in survival, with the greatest benefit occurring for interventions given prior to disease onset. Interventions at other time points were not significant, although this is likely to be secondary to a lack of publications examining these timepoints. The quality score had no impact on efficacy, and publication bias revealed an overestimation of the effect size, owing to one outlier study; excluding this led to the recalculated effect size changing from 5.31 to 3.31 (*P *<* *0.00001). The extant preclinical literature indicates that targeting mitochondrial dysfunction may prolong survival in amyotrophic lateral sclerosis, particularly if the intervention is administered early. A limitation of current research is a significant bias towards models based on superoxide dismutase 1, with uncertainty about generalisability to amyotrophic lateral sclerosis with an underlying TAR DNA binding protein 43 proteinopathy. However, further mechanistic research is clearly warranted in this field.

## Teaser

Here, we show a systematic review and meta-analysis of preclinical studies modelling amyotrophic lateral sclerosis and, specifically, examining mitochondrial dysfunction as a putative therapeutic target. Seventy-six studies were included, revealing that there is likely to be translational potential in targeting mitochondrial dysfunction, particularly if the intervention is delivered early.

## Graphical Abstract

**Graphical Abstract:**
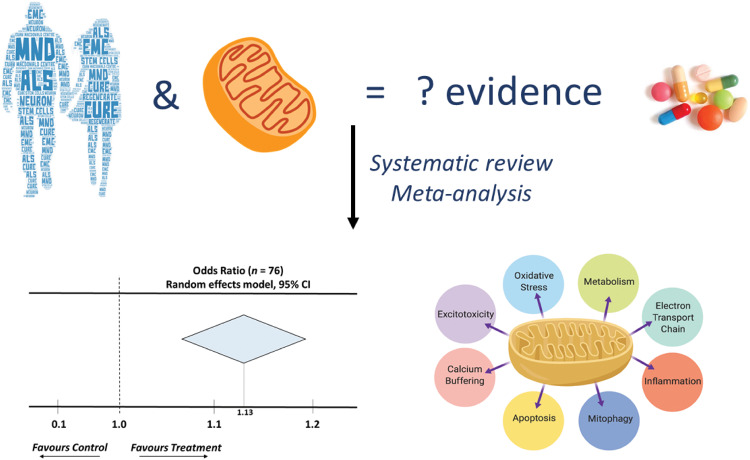

## Introduction

Amyotrophic lateral sclerosis (ALS), a type of motor neuron disease (MND), is a rapidly progressive, incurable and fatal neurodegenerative disorder, characterized by paralysis due to loss of upper and lower motor neurons (Brown and Al-Chalabi, 2017). There is no cure for ALS and only one globally licensed treatment (riluzole, first approved in 1995) that prolongs survival by a modest 2–3 months (Bensimon *et al.*, 1994; Miller *et al.*, 2012). The absence of effective disease modifying treatments, notwithstanding the promise of emerging candidates such as edaravone (Hardiman and van den Berg, 2017; The Writing Group and Edaravone (MCI-186) ALS 19 Study Group, 2017), highlights the need to address the unsuccessful translation of preclinical findings to the clinic (Mitsumoto *et al.*, 2014). A key requirement of advanced and accelerated drug discovery is that it must be informed by up-to-date, systematic, structured and unbiased analysis and review of the preclinical literature to enable both preclinical hypothesis generation and development of putative therapies for clinical trials.

ALS is associated with defects in energy metabolism (Vandoorne *et al.*, 2018) leading to weight loss, hypermetabolism and hyperlipidaemia (Dupuis *et al.*, 2011). Mitochondria are unique organelles, crucial for the regulation of metabolic pathways and cell survival. Through the Krebs cycle and the process of oxidative phosphorylation, nutrients from food that have been transported into a cell are converted into ATP, which is then used to fuel essential cellular processes. Alongside this canonical function of mitochondria, they also play a central role in calcium homeostasis and the regulation of apoptosis (Cozzolino and Carrì, 2012). Patients with ALS have dense clusters of mitochondria in the anterior horn of the lumbar spinal cord (Sasaki and Iwata, 1996) and presynaptic mitochondrial swelling in their motor neurons (Siklós *et al.*, 1996). The cellular distribution of mitochondria is also affected, with the majority of mitochondria in ALS neurons located in the soma and proximal axon (Sasaki *et al.*, 2007) and expression of Miro1, a protein that facilitates mitochondrial transport, is reduced in the spinal cord of ALS patients (Zhang *et al.*, 2015). Moreover, the total amount of mitochondrial DNA, measured by Southern blot, is reduced in the spinal cord from ALS patients (Wiedemann *et al.*, 2002). Dovetailing these changes, functional deficit has been demonstrated, insofar as patients with ALS have decreased activity of the electron transport chain complexes in spinal cord mitochondria (Wiedemann *et al.*, 2002) and the activity of key mitochondrial enzymes is reduced (Borthwick *et al.*, 1999). Notably, edaravone’s mechanism of action is thought to be via reducing oxidative stress. Furthermore, an extensive human induced pluripotent stem cell-based phenotypic screen of drugs using motor neurons derived from sporadic and familial cases identified ropinirole, a dopamine D2 receptor agonist, as the top candidate. Interestingly, its beneficial effect was attributed to it rescuing mitochondrial dysfunction, rather than its effects on dopamine signalling (Fujimori *et al.*, 2018). Finally, not only are there a plethora of ALS-causing mutations that have been identified that impact on mitochondrial function (Smith *et al.*, 2017), there is also direct evidence that disruption of mitochondrial structure and function contributes to the aetiology of ALS, owing to the discovery of causative mutations in the gene encoding the mitochondrial protein, CHCHD10, which is localized to contact sites between the inner and outer mitochondrial membrane (Bannwarth *et al.*, 2014).

Against this background, the primary aim of this review was to examine the animal model (preclinical) literature, and to evaluate the therapeutic potential of modulating mitochondrial pathways in ALS. The secondary aims were to: (i) determine the influence of timing of mitochondrial-based intervention and (ii) perform a quality assessment of the preclinical literature, including an assessment for publication bias. We hypothesized that interventions targeting mitochondrial dysfunction significantly affect survival in preclinical models of ALS.

## Materials and methods

The well-established framework of The Collaborative Approach to Meta-Analysis and Review of Animal Data from Experimental Studies (CAMARADES; www.camarades.info) was adopted in this study (Vesterinen *et al.*, 2014).

### Search methods

Preclinical data written in the English language were obtained, with no restrictions on publication date, from three databases: PubMed, Medline and EMBASE. The following search terms were used (search date: 21st March 2018):

#### PubMed

((‘motor neuron disease’ OR ‘motor neuron’ OR ‘MND’ OR ‘ALS’ OR ‘amyotrophic lateral sclerosis’) AND (‘mitochondria’) AND (‘mouse’ OR ‘mice’ OR ‘murine’ OR ‘rat’ OR ‘drosophila’ OR ‘fruit fly’ OR ‘c elegans’ OR ‘zebrafish’ OR ‘yeast’))

#### Medline

((‘motor neuron disease’ OR ‘motor neuron’ OR ‘MND’ OR ‘ALS’ OR ‘amyotrophic lateral sclerosis’) AND (‘mitochondria’) AND (‘mouse’ OR ‘mice’ OR ‘murine’ OR ‘rat’ OR ‘drosophila’ OR ‘fruit fly’ OR ‘c elegans’ OR ‘zebrafish’ OR ‘yeast’))

#### EMBASE

((‘motor neuron disease’ OR ‘motor neuron’ OR ‘MND’ OR ‘ALS’ OR ‘amyotrophic lateral sclerosis’) AND (‘mitochondria’) AND (‘mouse’ OR ‘mice’ OR ‘murine’ OR ‘rat’ OR ‘drosophila’ OR ‘fruit fly’ OR ‘c elegans’ OR ‘zebrafish’ OR ‘yeast’))

The references obtained from these searches were collated and imported into Endnote X8, where duplicate studies were removed and full-text articles were retrieved.

### Eligibility criteria

#### Inclusion criteria

Analysis of the effects of a therapeutic intervention on survival in an ALS disease model compared with a control group testing an intervention that targets mitochondria, including studies conducted in genetic or drug-induced models adopting the following model organisms: mouse, rat, *Drosophila*, zebrafish (*D. rerio*), *C. elegans* and yeast.

Noting that drugs can modulate multiple potential targets, and that the dogma of ‘single drug, single target’ is overly simplistic, here, for an intervention that targets mitochondria, we included any study stating an effect on this pathway, whether as a primary target or otherwise (Paolini *et al.*, 2006; Hopkins, 2008; Saha *et al.*, 2018). This was primarily to limit bias, with the aim of being comprehensive in our assessment of the literature. It is likely that the different therapeutic interventions identified would target multiple, potentially non-overlapping, pathways. The intention was that all interventions identified would have mitochondrial pathways as a commonality and, therefore, through meta-analysis of many such interventions, we would be able to identify a common therapeutic effect.

#### Exclusion criteria

We excluded: human trials, cell culture models, models where induction involved a combination of transgenesis and toxin; studies without a control group, publications or abstracts without potential for data extraction, and review articles, letters and comments.

### Screening

Screening for potentially relevant papers was completed via the Systematic Review Facility online screening tool (http://syrf.org.uk), a freely available, online systematic review tool. The title and abstract of each paper were screened by two independent reviewers against the eligibility criteria, and, in cases where reviewer concordance was <0.66, a third reviewer assessed the paper. The disposal of literature based on these criteria is duly presented in a PRISMA (Preferred Reporting Items for Systematic Reviews and Meta-Analyses) flowchart (Liberati *et al.*, 2009) and the included literature formed the analysis set.

### Data extraction

Our primary outcome measure was mortality. We extracted data for the following categories: intervention; efficacy demonstrated (statistically significant): Yes/No/Equivalent; survival in treatment group; survival in control group; number of individuals in treatment group; number of individuals in control group; timing of intervention: (i) prior to symptom onset, (ii) at symptom onset, (iii) after symptom onset and (iv) end-stage disease; model used; quality score based on modified CAMARADES criteria (Macleod *et al.*, 2004), with one point being given for each of the following (total score = 9): peer review publication, statement of temperature control, sample size calculation, appropriate control group identified, random allocation to treatment, allocation concealment, blinded assessment of outcome, compliance with animal welfare regulations, statement of potential conflict of interests.

### Statistical analysis

Survival data extracted from the included papers were included on a forest plot using the freely available Review Manager software (RevMan Version 5.3; Copenhagen: The Nordic Cochrane Centre, The Cochrane Collaboration, 2014). Given the variety of model organisms included in the analysis, we expressed effect sizes for the primary outcome data (survival summary data) as odds ratios for survival (Egger *et al.*, 2001; Vesterinen *et al.*, 2014). We calculated summary estimates within RevMan software using a random effects model, weighted by study size, via Hedges *G* statistic (Hedges and Olkin, 1985) to account for bias from small sample sizes. We reported statistical heterogeneity using *I*^2^ values (Higgins and Thompson, 2002), where an *I*^2^ of 0% represents no observed heterogeneity and larger values represent increasing heterogeneity, and we used funnel plotting to assess for the presence of publication bias. Predefined subgroup analyses were then performed to examine the effects of (i) timing of when the intervention was administered (intervention administered prior to the onset of symptoms, at symptom onset, after symptom onset); (ii) the pathways that each intervention acted on; (iii) the mode of delivery of the intervention; (iv) the target (cell-wide distribution or specific mitochondrial targeting); and (v) quality score, and the number of studies showing efficacy were compared between groups using a two-way ANOVA with Bonferroni correction for multiple comparisons (and chi^2^ for Fig. 4B and D). *Post hoc* sample size calculation was performed on studies included in the timing of intervention analysis. It was not possible to calculate an incidence from the latter two time points (after disease onset and end-stage disease); however, *post hoc* calculation of power performed on the second time point (intervention administered at disease onset) revealed that there was only a 12% power to detect a difference, confirming that there was an insufficient number of studies at this time point to make a definitive conclusion. Power calculation was performed as detailed below, performed on dichotomous outcomes using incidence of efficacy in each group compared to the population incidence (i.e. incidence in all studies).

$$\begin{array}{l} \text{Power}=\Phi \left\{\frac{\sqrt{N\text{*}\frac{\;{\left(P_{1}-P_{0}\right)}^{2}}{\left(P_{0}\text{ * }Q_{0}\right)}}-\;Z_{1}\;-\frac{\alpha }{2}\;}{\sqrt{\frac{\left(P_{1}\text{ * }Q_{1}\right)}{\left(P_{0}\text{ * }Q_{0}\right)}}}\right\}\\ \text{Power}=\Phi \left\{\frac{\sqrt{537*\frac{\;{\left(0.75-0.764\right)}^{2}}{\left(0.764\text{ * }0.236\right)}}-\;1.96}{\sqrt{\frac{\;\left(0.75\text{ * }0.25\right)}{\left(0.764\text{ * }0.236\right)}}}\right\}\\ \text{Power}=\Phi \left(-1.173\right)=0.12=12\text{\% power} \end{array}$$

### Data availability statement

All data have been made available through Supplementary material.

## Results

**Figure 1:**
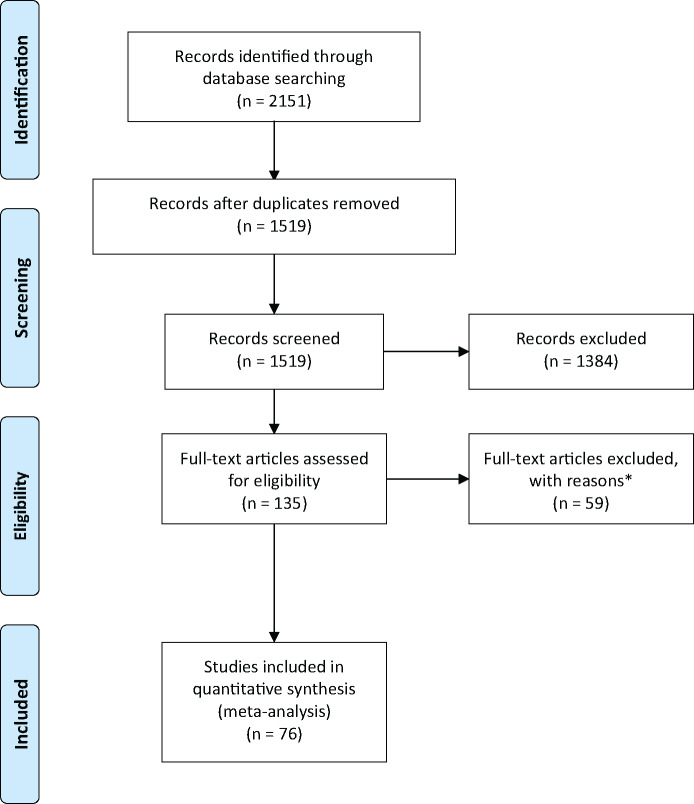
**PRISMA flowchart.** PRISMA (Preferred Reporting Items for Systematic Reviews and Meta-Analyses) flowchart indicating numbers of studies at each stage of the review. *Studies were excluded based on the following reasons: (i) due to inappropriate data formatting (i.e. where no useable data could be extracted); (ii) conference poster abstract with insufficient data; or (iii) inappropriately screened (i.e. where the study did not meet inclusion/exclusion criteria). For full dataset, see Supplementary Table 1.

Searches of PubMed, Medline and EMBASE yielded 2151 results. Following the removal of duplicates, 1519 remained. Of these 1519 studies, 1384 were excluded during the screening process and the full texts for the remaining 135 papers were obtained. During data extraction, a further 59 were excluded, owing to inappropriate or missing data. Thus, a total of 76 studies were included. Some studies assessed more than one pathway and/or drug; thus, 89 data points from 76 papers were included in the quantitative meta-analysis (Fig. 1; Supplementary Table 1). All the included studies adopted a rodent animal model, with most studies carried out in the *SOD1 G93A* mouse model (89%; Supplementary Fig. 1A).

### Meta-analysis reveals significant improvement in survival when targeting mitochondrial dysfunction

**Figure 2:**
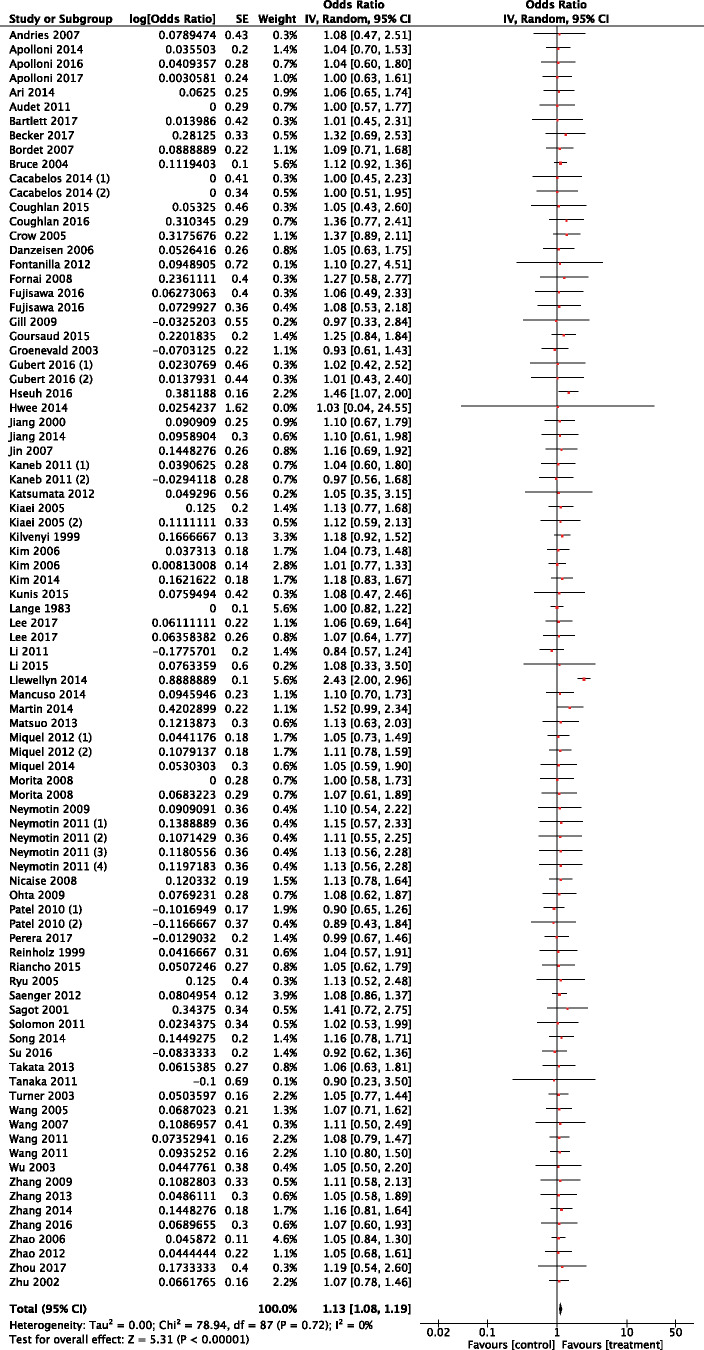
**Meta-analysis of 76 preclinical studies shows therapeutic potential for targeting mitochondrial pathways in ALS.** Forest plot showing the odds ratio and confidence intervals calculated from survival summary data from each study, weighted by study size. Overall effect estimate is demonstrated (with 95% confidence intervals) as a black diamond at the bottom of the graph. Heterogeneity is displayed as an *I*^2^ value. Results demonstrate an overall statistically significant effect favouring targeting of mitochondrial pathways in ALS.

Meta-analysis demonstrated that interventions targeting mitochondria are significantly more effective than control [test for overall effect estimate, *Z *=* *5.31 (*P *<* *0.00001); Fig. 2]. Furthermore, an assessment of heterogeneity resulted in an *I*^2^ value of 0%, demonstrating that there is no statistical heterogeneity between the studies included; thus, the result is a consistent finding. Having identified that the majority of the studies were carried out in the SOD1 mouse model, we also performed a *post hoc* subgroup analysis, using a random effects model and weighting as per Materials and methods section, on studies implementing models other than the SOD1 mouse model, including: the TAR DNA binding protein 43, *wobbler*, *pmn*, and valosin containing protein mouse models. Meta-analysis of these studies (*n *=* *5) favoured treatment (odds ratio = 1.39, *Z *=* *1.28, *P *=* *0.2) but this did not reach statistical significance (Supplementary Fig. 1B). However, *post hoc* calculation revealed that there was insufficient power to detect a difference, confirming that there were insufficient studies to make a definitive conclusion.

### Early manipulation of mitochondrial dysfunction results in improved survival

**Figure 3:**
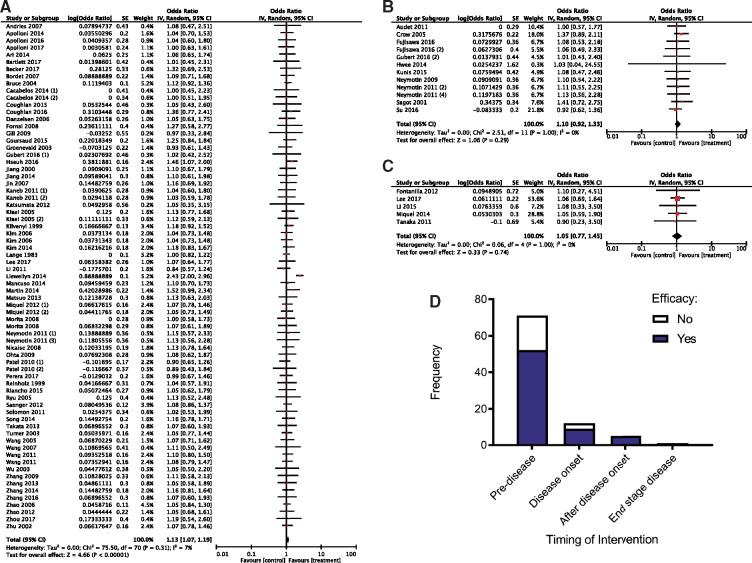
**The majority of studies are conducted at early time points and show that with early interventions there is a statistically significant improvement in survival.** Forest plots showing the odds ratio and confidence intervals calculated from survival summary data from each study, weighted by study size. Overall effect estimate is demonstrated (with 95% confidence intervals) as a black diamond at the bottom of the graph. Heterogeneity is displayed as an *I*^2^ value. (**A**) Interventions delivered pre-symptom onset; (**B**) at symptom onset and (**C**) after symptom onset. (**D**) Stacked frequency histogram illustrating the number of studies demonstrating efficacy (blue) as a proportion of the total number of studies (remaining white bar), divided into categories depending on timing of administered intervention. Results demonstrate an overall statistically significant effect favouring the targeting of mitochondrial pathways early in ALS (**A**); however, there is no difference in likelihood of demonstrating efficacy depending on timing of intervention (**D**), implying that there are too few studies conducted at later time points (two-way ANOVA, *P* > 0.05).

Secondary subgroup analysis examining the influence of timing of intervention on survival was conducted, grouping studies based on the time point at which the therapeutic intervention was administered (pre-symptom onset, at symptom onset and after symptom onset). Meta-analysis of studies conducted prior to symptom onset (Fig. 3A) revealed a statistically significant improvement in survival compared to controls (test of overall effect *Z *=* *4.66, *P *<* *0.00001) with an *I*^2^ value of 7%, indicating very little heterogeneity. However, when studies assessed after symptom onset were examined, there was no statistically significant improvement in survival, likely to be because there is a paucity of studies conducted examining these time points (Fig. 3B and C). Indeed, when studies were assessed for their probability of efficacy depending on whether the intervention was carried out prior to symptom onset or after symptom onset (all three time points, see Materials and methods section), there was no statistically significant effect (*P *>* *0.05; Fig. 3D). *Post hoc* power calculations performed on the first two time points (prior to symptom onset and at symptom onset) revealed that we had 100% and 12% power to detect a difference, respectively. It was not possible to calculate an incidence from the latter two time points (after disease onset and end-stage disease), since there were no negative studies reported in the small samples included; consequently, it was not possible to perform a *post hoc* power calculation. This analysis therefore revealed that there was an insufficient number of studies to make a definitive conclusion about the efficacy of interventions that were administered at later time points.

### Subgroup analyses reveal no difference in efficacy between divergent pathways, putative target or mode of delivery of intervention

**Figure 4:**
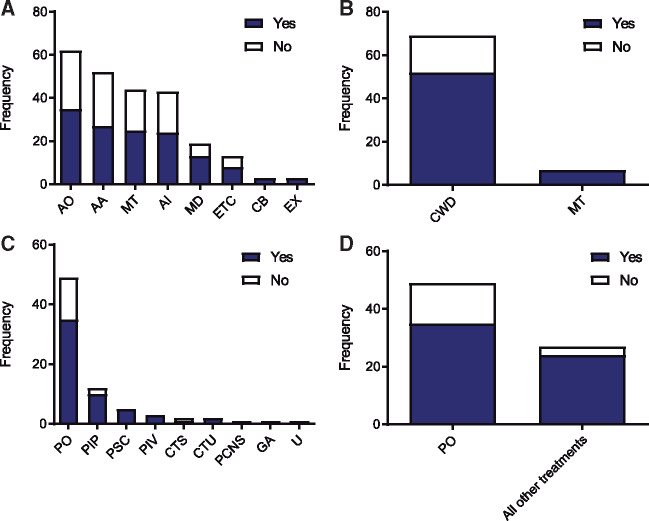
**Subgroup analyses reveal no difference in efficacy between divergent pathways, putative target or mode of delivery of intervention.** (**A**) Frequency of studies implementing therapeutic interventions that target the following pathways: AO, anti-oxidative stress; AA, anti-apoptosis; MT, metabolism; AI, anti-inflammation; MD, mitophagy/degradation; ETC, electron transport chain; CB, calcium buffering; EX, excitotoxicity. Each column shows studies demonstrating efficacy in blue and no efficacy in white. Two-way ANOVA shows no statistically significant difference (*P* > 0.05). (**B**) Frequency of studies implementing interventions that specifically target mitochondria (MT) versus more generic cell-wide distribution (CWD). Each column shows studies demonstrating efficacy in blue and no efficacy in white. Chi^2^ demonstrates no statistically significant difference (*P* = 0.14). (**C**). Frequency of the mode of delivery of study interventions: PO, pharmaceutical oral; PIP, pharmaceutical intraperitoneal; PSC, pharmaceutical subcutaneous; PIV, pharmaceutical intravenous; CTS, cell transplant specific cell type indicated; CTU, cell transplant no specific cell type indicated; PCNS, pharmaceutical directly administered to central nervous system; GA, genetic intervention, all cells targeted; U, unknown/not stated. Each column shows studies demonstrating efficacy in blue and no efficacy in white. Two-way ANOVA shows no statistically significant difference (*P* > 0.05). (**D**) *Post hoc* analysis performed to compare pharmaceutical oral (PO) versus all other modes of delivery grouped together. Chi^2^ demonstrates no statistically significant difference (*P* = 0.08).

Further subgroup analysis was performed to assess putative target pathways of the interventions tested in the analysis set (Fig. 4A). We identified eight target pathways: (i) anti-oxidative stress; (ii) anti-apoptosis; (iii) anti-inflammation; (iv) metabolism; (v) electron transport chain; (vi) mitophagy/degradation; (vii) calcium buffering; and (viii) excitotoxicity. Oxidative stress was the most frequently targeted pathway out of all interventions tested and there was no statistically significant difference in efficacy between interventions (*P *=* *0.08; Fig. 4A). Next, we analysed whether interventions were directly targeting mitochondria, or indirectly through a cell-wide distribution (Fig. 4B). Indeed, the majority of studies implemented interventions with a cell-wide distribution (91% of studies). 75% of studies with a cell-wide distribution demonstrated efficacy, compared with 100% of mitochondrially targeted interventions, although this difference did not reach statistical significance (*P *=* *0.136), likely because of the small number of studies implementing mitochondrially targeted therapies. Finally, we assessed the mode of delivery of interventions implemented in the analysis set (Fig. 4C). The most frequent mode of delivery was that of pharmacological agents delivered via the oral route, accounting for 64% of the studies included. The probability of demonstrating efficacy was lowest in this intervention category, with 71% of these studies showing efficacy, compared with 89% in all the other routes combined (Fig. 4D), although this difference did not reach statistical significance (*P *=* *0.08).

### Publication bias significantly overestimated the effect size

**Figure 5:**
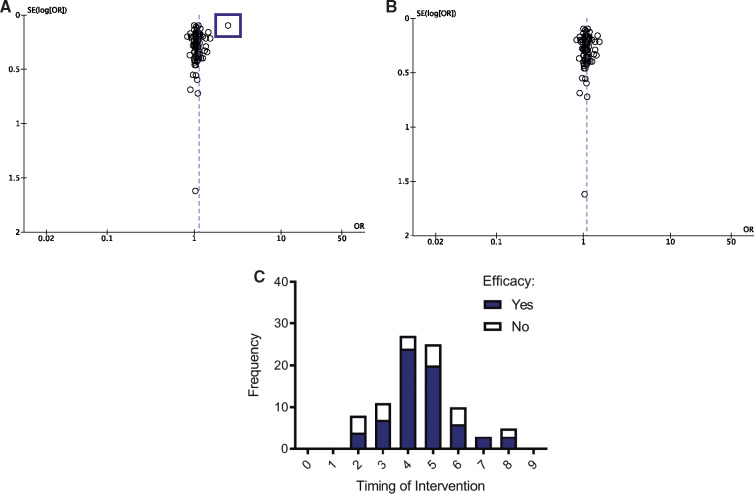
**Publication bias results in overestimation of effect size.** (**A**) Funnel plot showing each point as a study, plotted against the effect size of that study (abscissa) and precision of that study (SE(log[OR]); ordinate). One study outlier was identified (highlighted by a blue square), skewing the effect size significantly, resulting in an overestimation of the effect size. (**B**) Adjusted funnel plot demonstrating recalculated effect size estimate of 3.31, *cf*. 5.31.(**C**) Frequency distribution illustrating that the structured quality score is not significant in determining intervention efficacy (two-way ANOVA, *P* > 0.05).

Funnel plot demonstrated publication bias (Fig. 5). An outlier (lying more than two standard deviations from an otherwise tightly distributed dataset) was noted in the dataset (Fig. 5A). To investigate this further, the outlier was disregarded in a *post hoc* analysis and the funnel plot was accordingly replotted (Fig. 5B). The recalculated effect size can now be seen to pass directly through the cluster that was previously sitting to the left of the line; thus, the effect size decreases from 5.31 to 3.31. Whilst still statistically significant, this outlier is likely contributing to an overestimation of the estimated effect size of mitochondrial interventions. Furthermore, a structured quality assessment was performed on the included studies; the majority of papers scored highly in certain categories, for example: (i) peer-reviewed articles; (ii) identification of appropriate control groups; and (iii) statements regarding conflicts of interest. However, other important categories were not so well-represented. The most strikingly absent criterion was the use of a sample size calculation which, although one of the pivotal aspects of designing a study, was used in only 2 out of 76 papers. Despite this, two-way ANOVA showed that the quality of the research did not significantly impact the efficacy of the interventions being tested (Fig. 5C;Supplementary Table 1).

## Discussion

In summary, the data show that there is likely to be potential in targeting mitochondrial dysfunction in ALS (test for overall effect *Z *=* *5.31, *P *<* *0.00001), with particular strength for interventions administered prior to disease onset (test for overall effect *Z *=* *4.66, *P *<* *0.00001). These findings are promising, since they have the potential to form not only the basis of future treatments for ALS, but they also encourage mechanistic study focused on mitochondrial dysfunction, because of the inference that this is likely to be an early, perhaps presymptomatic, pathological event. Before discussing the implications of our findings, it is worth considering the strengths and weaknesses of our approach, and the limitations of the findings.

A major strength of our overview approach is that this is the first explicit systematic review, meta-analysis and structured quality assessment of the preclinical ALS literature examining the influence of interventions targeting mitochondrial dysfunction on survival. Preclinical studies underpin our understanding of disease mechanisms and are crucial for testing interventions for safety and efficacy. However, they come with the caveat that animal studies are inherently heterogeneous—much more so than a clinical trial (Vesterinen *et al.*, 2014)—and so they are useful only if heterogeneity and their impact on the effect size are taken into consideration. Accordingly, our approach, combining systematic review and meta-analysis, addresses this.

The main weakness stems from the eligibility criteria, by virtue of the fact that studies not reporting alterations in survival were excluded. Thus, following the screening process, 135 studies were included in the meta-analysis. However, only 76 of these studies investigated survival in their models used and, therefore, 59 papers were automatically excluded. It would be desirable to incorporate alternative outcome measures (such as, for example, motor neuron counts, molecular biomarkers, body mass, rotarod performance, gait analysis and behavioural outcomes) but they need to be fully characterized, quantified and reported in the model. Such outcomes have the potential to translate into surrogates of quality of life outcomes relevant to people with ALS and their caregivers.

### Study limitations

First, the approach to determine potential therapeutic interventions was based on the identification of a statement by the authors in the included papers that the intervention targeted putative mitochondrial pathways. This method was employed to limit bias and to identify as comprehensive a dataset as possible, being cognizant that most drugs will have multiple target pathways, and it assumes that those identified will have mitochondrial targeting as a commonality. Notably, no approach to identifying such interventions is perfect, because of, sometimes unknown or not reported, off-target effects of drugs. If authors did not mention mitochondrial targeting in their study, we were not able to include it in our analysis. An example of this limitation would be studies examining the effects of rapamycin. Rapamycin is a drug known mostly for its effect on autophagy; however, rapamycin has also been shown to have multiple targets, including effects on mitochondrial dysfunction, primarily through its role in mitophagy, cellular metabolism and apoptosis (Garza-Lombó *et al.*, 2018; Li *et al.*, 2018; Johnson *et al.*, 2019). Thus, as a result of its divergent roles in different molecular pathways, and our inclusion criteria aimed at limiting bias, not all studies assessing the effect of rapamycin will have been included—only those where the authors explicitly mentioned a role in mitochondrial pathways.

Second, we show that the overall quality of the studies conducted in the preclinical field of mitochondrial dysfunction in ALS is poor. Using a modification of the CAMARADES criteria (Macleod *et al.*, 2004), an established quality score for assessing overall methodological quality of individual studies in the preclinical literature, a structured quality assessment was performed for each paper included in the meta-analysis. The majority of papers scored highly in certain categories, for example: (i) peer-reviewed articles, (ii) identification of appropriate control groups and (iii) statements regarding conflicts of interest. However, other important categories were not so well-represented. The most strikingly absent criterion was the use of a sample size calculation which, although one of the pivotal aspects of designing a study, was used in only 2 out of 76 papers. Since it is impossible to study a whole population, it is necessary to obtain a fully representative sample using a sample size calculation. Without it, a sample may be too small to detect a significant result, or too large, with consequent ethical and financial implications. Furthermore, if riluzole, which has been shown to have a limited, but significant, effect in prolonging survival in patients with ALS (Miller *et al.*, 2012), is used as a benchmark positive control compound in the most widely published mouse model of ALS, assuming a modest effect size (*circa* 1%), even studies of *n *>* *50 animals per cohort would be insufficiently powered to detect it (Scott *et al.*, 2008). Given that the sample sizes in the studies included in the meta-analysis ranged from 4 to 39 (median = 13), it is likely that the vast majority, if not all, of these were underpowered. There was also a distinct lack of random allocation and blinding in a large number of the studies, subjecting these and their effect sizes to unknown and unnecessary bias. An important initiative set out with the aim to replicate several studies published in the preclinical domain in ALS found that there was consistent overestimation of the true effect size (Perrin, 2014). It is therefore possible that the overall effect size calculated in our study may be over-inflated, due to widespread overestimation of the effect size in studies particularly predating 2010, when guidelines were drawn up (Ludolph *et al.*, 2010).

Third, out of the 76 studies that were included in the meta-analysis, 89% used *SOD1 G93A* transgenic mouse models, where there is overexpression (usually 23 copies) of human genomic mutant *SOD1* (Gurney *et al.*, 1994). We encountered difficulties from the lack of reporting the transgene copy number and death from non-ALS causes in the studies incorporated in our review. Seventeen studies stated that they used the high copy number mouse model; 2 studies stated that they used the low copy number mouse model and 50 (72% of the included studies) did not state the model used. Both of these variables affect the conclusions that one can reliably draw, given that it is well known that high copy number models show faster disease progression and earlier death compared with those adopting a low copy number mice (Turner and Talbot, 2008), and these mice may have a heightened susceptibility to infections and other non-ALS related illnesses (Scott *et al.*, 2008; van der Worp *et al.*, 2010). Although this transgenic mouse model was and remains important to the understanding of disease mechanisms (Van Damme *et al.*, 2017), the over-representation of studies using this model needs to be considered; furthermore, whilst our subgroup analysis of non-SOD1 model studies revealed no significant difference in therapies targeting mitochondrial pathways, there was an insufficient number of studies to make a definitive conclusion at this time. The *SOD1* mutation accounts for a minority (<2%) of ALS patients (Rosen *et al.*, 1993) and, importantly, *SOD1* cases do not display the pathological hallmark of ALS, namely TAR DNA binding protein 43 proteinopathy, that is found in >97% of ALS cases (Neumann *et al.*, 2006; Kwiatkowski *et al.*, 2009). Thus, *SOD1* transgenic models are likely to recapitulate only certain aspects of ALS (Joyce *et al.*, 2011); notwithstanding this, there is emerging evidence that misfolded SOD1 protein is pathologically present in sporadic ALS with no known genetic cause (Paré *et al.*, 2018), and in cases of ALS caused by mutations in known ALS genes other than *SOD1* (Forsberg *et al.*, 2019), suggesting that SOD1 biology may well be involved in ALS pathogenesis in general. With respect to mitochondrial neurobiology, aberrations in mitochondrial morphology, function, clearance and transport have been observed in mutant SOD1 mice (Shi *et al.*, 2010).

Finally, whilst there is substantial homology between animal models and humans in terms of conserved genes, organ systems and systemic physiology, there are also many important differences. Indeed the mitochondrial genome, a double-stranded closed-circular molecule, has substantial homology between human and rodent species, with 16 569 base pairs in humans and 16 301 base pairs in the mouse (Wallace and Fan, 2009). However, there are important functional differences at an organismal level (Uhl and Warner, 2015), and in ageing (Demetrius, 2006) and metabolism (Svenson *et al.*, 2007), including fundamental differences in the production rate of reactive oxygen species between mice and humans (Finkel and Holbrook, 2000). A well-established manifestation of such a difference relates to the influence of calorie restriction on phenotypic measures of mitochondrial function. Several preclinical studies have demonstrated, via calorie restriction, a protective effect on survival, through the upregulation of protective mitochondrial pathways associated with apoptosis, reduced reactive oxygen species and an overall reduction in metabolic demand. Indeed, calorie restriction has been shown to extend lifespan in many laboratory organisms and plays a preventative role in ageing and diseases, including diabetes and cardiovascular diseases (Partridge and Brand, 2005; Fontana *et al.*, 2008; Piper *et al.*, 2011). However, a large systematic review and meta-analysis of dietary restriction in the animal model literature concluded that this is more likely to be the result of adaption to laboratory conditions, rather than evolutionary conservation (Nakagawa *et al.*, 2012). Indeed, evidence for translation of the protective effects of calorie restriction to humans is poor (Phelan and Rose, 2005). It is therefore possible that pathways identified through this review may demonstrate efficacy in animal models, but that their translation to human therapeutics is limited, highlighting the need for further evaluation in human-based models.

### Putative molecular pathways

The most targeted pathway in the extant preclinical literature is the anti-oxidative stress pathway. However, no pathway has been shown to be better than any others and no intervention has been tested more than three times, questioning the reproducibility of findings. We suggest that this is likely to be in part as a result of the research tools that have been used to detect putative targets (such as the cleaved caspase 3 assay), rather than pure specificity of the target *per se*. Some pathways are under-represented, such as studies examining for defects in the autophagic turnover of damaged mitochondria, termed mitophagy, despite their being recent advances in tools to examine both mitophagy and mitochondrial architecture simultaneously *in vivo*, such as *mito*-QC (McWilliams *et al.*, 2016). There is also a paucity of literature specifically exploring the influence of aberrant mitochondrial pathways in glia *in vivo*, including their dynamics (Jackson and Robinson, 2018). Interestingly, there were very few studies adopting interventions that were specifically targeted to the mitochondria, for instance, through conjugation to the lipophilic cation, triphenyl phosphonium (Zielonka *et al.*, 2017), with the majority of studies adopting interventions that had a cell-wide distribution. The latter is a contributor to the limited success of interventions, despite their promising theoretical mechanism of action, because only a fraction of the compound is taken up by the mitochondria.

### Mode of administration of intervention

We show that oral administration was the most frequent mode of intervention and there was a trend towards greater efficacy in studies adopting other routes of administration. This most likely reflects the poor oral bioavailability of oral medications and has been cited as a major flaw in previously conducted clinical trials in the ALS field (Mitsumoto *et al.*, 2014), notwithstanding the difficulties many patients face with swallowing, owing to brainstem dysfunction (Brown and Al-Chalabi, 2017).

### Implications for future research

Through conducting this systematic review and meta-analysis, we conclude that targeting mitochondrial dysfunction in ALS is likely to hold therapeutic potential and that further research should be carried out to delve into the mechanisms underlying the deficit and to further investigate the optimal timing of intervention. There is clearly a focus in the current literature on presymptomatic treatment of animals, which makes these therapies more difficult to translate to the clinic. Whilst genetic testing could identify individuals with potential for presymptomatic intervention, the majority of ALS cases are not caused by a known genetic mutation. Furthermore, there is no existing biomarker for the detection of early disease states in these cases. We therefore recommend that preclinical studies focus on interventions at or after symptom onset to improve the translational potential of these studies. We also recommend that preclinical research should adopt a wide variety of models (Van Damme *et al.*, 2017; Lutz, 2018), particularly of mutations accounting for the commoner causes of ALS, such as the *C9orf72* repeat expansion (DeJesus-Hernandez *et al.*, 2011; Renton *et al.*, 2011). Each model must be fully characterized and suitably powered, and a range of outcome measures reported, in addition to survival, at various time points, particularly later time points following the onset of symptoms, when interventions are likely to have the greatest translational benefit and where there currently is a paucity of studies. Pharmacological compounds ought to be mitochondrially targeted, with thought given to identifying a suitable route of administration that maximizes bioavailability, and coupled to a more comprehensive examination of their effects on multiple mitochondrial signalling and molecular pathways. Finally, technological advances in human induced pluripotent stem cells and gene editing offer unprecedented opportunities to develop new experimental human models of monogenic neurological diseases (Sandoe and Eggan, 2013). Human induced pluripotent stem cells maximize human relevance, mechanistic insight and also afford high throughput (Dolmetsch and Geschwind, 2011). They uniquely facilitate the study of the non-cell-autonomous influences of mitochondrial gene transcription, owing to the ability to co-culture mixed species-derived neurons with human induced pluripotent stem cell-derived astrocytes (for example) and the identification of signalling pathways through *in silico* RNA sequencing read sorting (Qiu *et al.*, 2018). Human induced pluripotent stem cells also permit the study of live cells in assays examining disease mechanistic vulnerability and sufficiency, and, as such, we recommend that researchers adopt them to focus on investigating perturbations in mitochondrial pathways, adhering to recent published guidelines (Connolly *et al.*, 2018), thereby expanding the repertoire of ALS disease models.

## Funding

A.R.M. is a Lady Edith Wolfson Clinical Fellow and is jointly funded by the Medical Research Council and the Motor Neurone Disease Association (MR/R001162/1). He also acknowledges support from the Rowling Scholars scheme, administered by the Anne Rowling Regenerative Neurology Clinic. Both the Chandran and Hardingham laboratories are supported by the Euan MacDonald Centre and the UK Dementia Research Institute partner funders: the Medical Research Council, Alzheimer’s Research UK and the Alzheimer’s Society. J.M.G is funded by a starter grant for clinical lecturers from the Academy of Medical Sciences (AMS: 210JMG 3102 R45620).

## Competing interests

The authors report no competing interests.

## Supplementary Material

fcz009_Supplementary_Data

## Abbreviations

ALS =: amyotrophic lateral sclerosis
C9orf72 =: chromosome 9 open reading frame 72
CAMARADES =: The Collaborative Approach to Meta-Analysis and Review of Animal Data from Experimental Studies
CHCHD10 =: Coiled-coil-helix-coiled-coil-helix domain containing 10
Miro1 =: mitochondrial Rho GTPase 1
MND =: motor neuron disease
SOD1 =: superoxide dismutase 1
pmn =: progressive motor neuronopathy
PRISMA =: Preferred Reporting Items For Systematic Reviews and Meta-analyses
TDP-43 =: TAR DNA binding protein 43
VCP =: valosin containing protein
