# Supplementary material for: Targeting mitochondrial dysfunction in amyotrophic lateral sclerosis: a systematic review and meta-analysis
Source: Brain Commun. 2019 Aug 6;1(1):fcz009. doi: 10.1093/braincomms/fcz009 (PMC7056361; doi:10.1093/braincomms/fcz009)
Supplement: fcz009_Supplementary_Data [file fcz009_supplementary_data.zip › BRAINCOM-2019-020_Manuscript_Original_submission.pdf]

OXFORD UNIVERSITY PRESS | Brain Communications

Targeting mitochondrial dysfunction in amyotrophic lateral sclerosis – a systematic review and meta-analysis of preclinical research

Journal:	*Brain Communications*
Manuscript ID	BRAINCOM-2019-020
Manuscript Type:	Review Article
Date Submitted by the Author:	09-Apr-2019
Complete List of Authors:	Mehta, Arpan; University of Edinburgh College of Medicine and Veterinary Medicine, Centre for Clinical Brain Sciences<br>Walters, Rachel; University of Edinburgh College of Medicine and Veterinary Medicine, Centre for Clinical Brain Sciences<br>Waldron, Fergal; The University of Edinburgh Institute of Evolutionary Biology, Institute of Evolutionary Biology<br>Pal, Suvankar; University of Edinburgh College of Medicine and Veterinary Medicine, Centre for Clinical Brain Sciences<br>Selvaraj, Bhuvaneish; University of Edinburgh College of Medicine and Veterinary Medicine, Centre for Clinical Brain Sciences<br>MacLeod, Malcolm; University of Edinburgh College of Medicine and Veterinary Medicine, Centre for Clinical Brain Sciences<br>Hardingham, Giles; University of Edinburgh College of Medicine and Veterinary Medicine, Centre for Clinical Brain Sciences<br>Chandran, Siddharthan; University of Edinburgh College of Medicine and Veterinary Medicine, Centre for Clinical Brain Sciences<br>Gregory, Jenna; University of Edinburgh College of Medicine and Veterinary Medicine, Centre for Clinical Brain Sciences
Keywords:	Systematic review, Meta-analysis, Mitochondria, Amyotrophic lateral sclerosis, Preclinical

SCHOLARONE™ Manuscripts

Targeting mitochondrial dysfunction in amyotrophic lateral sclerosis – a systematic review and meta-analysis of preclinical research

Arpan R. Mehta[1,2,3,4,5], Rachel Walters[2,3,4], Fergal M. Waldron[6], Suvankar Pal[2,3,4], Bhuvaneish T. Selvaraj[1,2,4], Malcolm R. Macleod[2,3,4], Giles E. Hardingham[1,4,7], Siddharthan Chandran[1,2,3,4,5,8,9,10]* and Jenna M. Gregory[1,2,3,4,11]*

[1] UK Dementia Research Institute, The University of Edinburgh, Edinburgh, UK.

[2] Centre for Clinical Brain Sciences, The University of Edinburgh, Edinburgh, UK.

[3] The Anne Rowling Regenerative Neurology Clinic, The University of Edinburgh, Edinburgh, UK.

[4] The Euan MacDonald Centre, University of Edinburgh, Edinburgh, UK.

[5] Nuffield Department of Clinical Neurosciences, University of Oxford, Oxford, UK.

[6] Institute of Evolutionary Biology and Centre for Immunity Infection and Evolution, University of Edinburgh, Ashworth Laboratories, Charlotte Auerbach Road, Edinburgh, UK.

[7] Centre for Discovery Brain Sciences, The University of Edinburgh, Edinburgh, UK.

[8] Centre for Brain Development and Repair, inStem, Bangalore, India.

[9] MRC Centre for Regenerative Medicine, The University of Edinburgh, Edinburgh, UK.

[10] Edinburgh Neuroscience, The University of Edinburgh, Edinburgh, UK.

[11] MRC Edinburgh Brain Bank, Academic Department of Neuropathology, The University of Edinburgh, Edinburgh, UK.

***Denotes co-corresponding author**

For Review Only

Word count: 3863 (including references)

References: 39

Abstract

Objectives

Interventions targeting mitochondrial dysfunction have the potential to extend survival in preclinical models of amyotrophic lateral sclerosis (ALS). The aim of this systematic review was to assess the efficacy of targeting mitochondria as a potential therapeutic target in ALS.

Methods

Preclinical studies written in the English language were identified with no restrictions on publication date from PubMed, Medline and EMBASE databases. All studies adopting interventions targeting mitochondria to treat ALS in genetic or drug-induced organism models were considered for inclusion. A total of 88 studies were included in the analysis. Survival data were extracted and the meta-analysis was completed in RevMan 5 software.

Results

Targeting mitochondrial dysfunction in ALS results in a statistically significant improvement in survival ($Z = 5.31$; $P < 0.00001$). The timing of administration of the intervention appears to affect the improvement in survival, with the greatest benefit occurring in interventions given prior to disease onset. Interventions at other time points were not significant, although this is likely to be secondary to a lack of publications examining these timepoints. The quality score had no impact on efficacy, and publication bias revealed an overestimation of the effect size owing to one outlier study; excluding this led to the recalculated effect size changing from 5.31 to 3.31 ($P < 0.00001$).

Conclusions

The extant preclinical literature indicates that targeting mitochondrial dysfunction prolongs survival in ALS, particularly if the intervention is administered early. Further mechanistic research is clearly warranted in this field.

For Review Only

Introduction

Amyotrophic lateral sclerosis (ALS), a type of motor neurone disease (MND), is a rapidly progressive, incurable, and fatal neurodegenerative disorder, characterized by paralysis due to loss of upper and lower motor neurons[1]. There is no cure for ALS and only one globally licensed treatment (Riluzole, first approved in 1995) that prolongs survival by a modest 2-3 months. The absence of effective disease modifying treatments, notwithstanding the promise of emerging candidates such as edaravone[2], highlights the need to address the unsuccessful translation of pre-clinical findings to the clinic. A key requirement of advanced and accelerated drug discovery is that it must be informed by up-to-date, systematic, structured and unbiased analysis and review of the pre-clinical literature to enable both preclinical hypothesis generation and development of putative therapies for clinical trials.

ALS is associated with defects in energy metabolism[3] leading to weight loss, hypermetabolism and hyperlipidaemia [4]. Mitochondria are unique organelles, crucial for the regulation of metabolic pathways and cell survival. Through the Krebs Cycle and the process of oxidative phosphorylation, nutrients from food that have been transported into a cell are converted into ATP, which is then used to fuel essential cellular processes. Alongside this canonical function of mitochondria, they also play a central role in calcium homeostasis and the regulation of apoptosis[5]. Patients with ALS have dense clusters of mitochondria in the anterior horn of the lumbar spinal cord[6] and presynaptic mitochondrial swelling in their motor neurons[7]. The cellular distribution of mitochondria is also affected, with the majority of mitochondria, in ALS neurons, located in the soma and proximal axon and expression of Miro1, a protein that facilitates mitochondrial transport, is reduced in the spinal cord of ALS patients[8]. Moreover, the total amount of mitochondrial DNA, measured by Southern blot, is reduced in the spinal cord from ALS patients[9]. Dovetailing these changes, functional deficit has been demonstrated, insofar as patients with ALS have decreased activity of the electron transport chain complexes in spinal cord mitochondria[10] and the activity of key mitochondrial enzymes is reduced[11]. Interestingly, edaravone's mechanism of action is thought to be via reducing oxidative stress. Furthermore, an extensive human induced pluripotent stem cell based phenotypic screen of drugs using motor neurons derived from sporadic and familial cases identified ropinirole, a dopamine D2 receptor agonist, as the top

For Review Only

candidate. Interestingly, its beneficial effect was attributed to rescuing mitochondrial dysfunction rather than its effects on dopamine signaling[12].

Against this background, the primary aim of this review was to review the animal model (preclinical) literature to evaluate the therapeutic potential of modulating mitochondrial pathways in ALS. The secondary aims were to: (i) determine the influence of timing of mitochondrial based intervention (ii) perform a quality assessment of the preclinical literature, including an assessment for publication bias. We hypothesised that interventions targeting mitochondrial dysfunction significantly affect survival in preclinical models of ALS.

Methods

The well-established framework of The Collaborative Approach to Meta-Analysis and Review of Animal Data from Experimental Studies (CAMARADES; www.camarades.info) was adopted in this study[13].

Search Methods

Preclinical data written in the English language were be obtained, with no restrictions on publication date, from three databases: PubMed, Medline and EMBASE. The following search terms were used (search date - 21st March 2018):

PubMed

(("motor neuron disease" OR “motor neuron” OR “MND” OR "ALS" OR "amyotrophic lateral sclerosis") AND ("mitochondria") AND ("mouse" OR "mice" OR "murine" OR "rat" OR “drosophila” OR “fruit fly” OR “c elegans” OR “zebra fish” OR “yeast”))

Medline

(("motor neuron disease" OR “motor neuron” OR “MND” OR "ALS" OR "amyotrophic lateral sclerosis") AND ("mitochondria") AND ("mouse" OR "mice" OR "murine" OR "rat" OR “drosophila” OR “fruit fly” OR “c elegans” OR “zebra fish” OR “yeast”))

EMBASE

For Review Only

(("motor neuron disease" OR “motor neuron” OR “MND” OR "ALS" OR "amyotrophic lateral sclerosis") AND ("mitochondria") AND ("mouse" OR "mice" OR "murine" OR "rat" OR “drosophila” OR “fruit fly” OR “c elegans” OR “zebra fish” OR “yeast”))

The references obtained from these searches were collated and imported into Endnote X8, where duplicate studies were removed and full text articles were retrieved.

Eligibility criteria

Inclusion Criteria

Analysis of the effects of a therapeutic intervention on survival in an ALS disease model compared with a control group testing an intervention that targets mitochondria, including studies conducted in genetic or drug-induced models adopting the following model organisms: mouse, rat, *Drosophila*, zebrafish (*D. rerio*), *C. elegans* and yeast.

Exclusion Criteria

We excluded: human trials, cell culture models, models where induction involved a combination of transgenesis and toxin; studies without a control group, publications or abstracts without potential for data extraction, and review articles, letters and comments.

Screening

Screening for potentially relevant papers was completed via the Systematic Review Facility online screening tool (http://syrf.org.uk), a freely available, online systematic review tool. The title and abstract of each paper were screened by two independent reviewers against the eligibility criteria, and, in cases where reviewer concordance was <0.66, a third reviewer assessed the paper. The disposal of literature based on these criteria were duly presented in a PRISMA flowchart and the included literature formed the analysis set.

Data extraction

Our primary outcome measure was mortality. We extracted data for the following categories: Intervention; efficacy demonstrated (statistically significant): Yes/No/Equivalent; survival in treatment group; survival in control group; number of individuals in treatment group; number of individuals in control group; timing of intervention: prior to symptom onset (1), at symptom

For Review Only

onset (2), after symptom onset (3), end-stage disease (4); model used; quality score based on modified CAMARADES criteria[14], with one point being given for each of the following (total score = 9): peer review publication, statement of temperature control, sample size calculation, appropriate control group identified, random allocation to treatment allocation concealment, blinded assessment of outcome, compliance with animal welfare regulations, statement of potential conflict of interests.

Data analysis

Survival data extracted from the included papers were included on a forest plot using the freely available Review Manager software (RevMan Version 5.3; Copenhagen: The Nordic Cochrane Centre, The Cochrane Collaboration, 2014). Given the variety of model organisms included in the analysis, we expressed effect sizes for the primary outcome data (survival summary data) as odds ratios for survival[13]. We calculated summary estimates within RevMan software using a random effects model, weighted by study size, via Hedges *G* statistic to account for bias from small sample sizes. We reported statistical heterogeneity using I^2 values, where an I^2 of 0% represents no observed heterogeneity and larger values represent increasing heterogeneity, and we used funnel plotting to assess for the presence of publication bias. Predefined subgroup analyses were then performed to examine the effects of (i) timing of when the intervention was administered (intervention administered prior to the onset of symptoms, at symptom onset, after symptom onset); (ii) the pathways that each intervention acted on; (iii) the mode of delivery of the intervention; (iv) the target (cell-wide distribution or specific mitochondrial targeting); and (v) quality score, and the number of studies showing efficacy were compared between groups using a 2-way ANOVA with Bonferroni correction for multiple comparisons (and chi^2 for Figures 4B and 4D). *Post hoc* sample size calculation was performed on studies included in the timing of intervention analysis. It was not possible to calculate an incidence from the latter two time points (after disease onset and end-stage disease); however, *post hoc* calculation of power performed on the second time point (intervention administered at disease onset) revealed that there was only a 12 % power to detect a difference, confirming that there was an insufficient number of studies at this time point to make a definitive conclusion. Power calculation was performed as detailed below, performed on dichotomous outcomes using incidence of efficacy in each group compared to the population incidence (*i.e.,* incidence in all studies).

$$Power = \Phi\left\{\frac{\sqrt{N * \frac{(P_1-P_0)^2}{(P_0*Q_0)}} - z_{1-\alpha/2}}{\sqrt{\frac{P_1*Q_1}{(P_0*Q_0)}}}\right\}$$

$$Power = \Phi\left\{\frac{\sqrt{537 * \frac{(0.75-0.764)^2}{(0.764*0.236)}} - 1.96}{\sqrt{\frac{0.75*0.25}{(0.764*0.236)}}}\right\}$$

$$Power = \Phi(-1.173) = 0.12 = 12\%\ power$$

Results

Searches of PubMed, Medline and EMBASE yielded 2151 results. Following the removal of duplicates, 1519 remained. Of these 1519 studies, 1384 were excluded during the screening process and the full texts for the remaining 135 papers were obtained. During data extraction, a further 47 were excluded, owing to inappropriate or missing data. Thus, a total of 88 studies were included in the quantitative meta-analysis (Figure 1; supplemental table 1). All the included studies adopted a rodent animal model, with most studies carried out in the *SOD1 G93A* mouse model (86%).

For Review Only

Meta-analysis reveals significant improvement in survival when targeting mitochondrial dysfunction

Meta-analysis demonstrated that interventions targeting mitochondria are significantly more effective than control (test for overall effect estimate, Z = 5.31 ($P < 0.00001$); Figure 2). Furthermore, an assessment of heterogeneity resulted in an I^2 value of 0%, demonstrating that there is no statistical heterogeneity between the studies included; thus the result is a consistent finding.

Early manipulation of mitochondrial dysfunction results in improved survival

Secondary subgroup analysis examining the influence of timing of intervention on survival was conducted, grouping studies based on the time point at which the therapeutic intervention was administered (pre-symptom onset, at symptom onset, and after symptom onset). Meta-analysis of studies conducted prior to symptom onset (Figure 3A) revealed a statistically significant improvement in survival compared to controls (test of overall effect Z = 4.66 ($P < 0.00001$)) with an I^2 value of 7%, indicating very little heterogeneity. However, when studies assessed after symptom onset were examined, there was no statistically

significant improvement in survival (Figures 3B&3C), likely to be because of fewer studies conducted examining these time points (Figures 3B&3C). Indeed, when studies were assessed for their probability of efficacy depending on whether the intervention was carried out prior to symptom onset or after symptom onset (all three time points, see Methods), there was no statistically significant effect (P>0.05; Figure 3D). *Post hoc* power calculations performed on the first two time points (prior to symptom onset and at symptom onset) revealed that we had 100 % and 12 % power to detect a difference, respectively. It was not possible to calculate an incidence from the latter two time points (after disease onset and end stage disease), since there were no negative studies reported in the small samples included; consequently, it was not possible to perform a *post hoc* power calculation. This analysis therefore revealed that there was an insufficient number of studies to make a definitive conclusion about the efficacy of interventions that were administered at later time points.

Subgroup analyses reveal no difference in efficacy between divergent pathways, putative target or mode of delivery of intervention

Further subgroup analysis was performed to assess putative target pathways of the interventions tested in the analysis set (Figure 4A). We identified 8 target pathways: (i) antioxidative stress (AO); (ii) anti-apoptosis (AA); (iii) anti-inflammation (AI); (iv) metabolism (MT); (v) electron transport chain (ETC); (vi) mitophagy/degradation (MD); (vii) calcium buffering (CB); and (viii) excitotoxicity (EX). Oxidative stress was the most frequently targeted pathway out of all interventions tested and there was no statistically significant difference in efficacy between interventions (P=0.08; Figure 4A). Next, we analysed whether interventions were directly targeting mitochondria, or indirectly through a cell-wide distribution (Figure 4B). Indeed, the majority of studies implemented interventions with a cell-wide distribution (91% of studies). 75% of studies with a cell wide distribution demonstrated efficacy, compared to 100% of mitochondria-targeted interventions, although this difference did not reach statistical significance (P=0.136), likely because of the small number of studies implementing mitochondrially-targeted therapies. Finally, we assessed the mode of delivery of interventions implemented in the analysis set (Figure 4C). The most frequent mode of delivery was that of pharmacological agents delivered via the oral route, accounting for 64% of the studies included. The incidence of efficacy was lowest in this intervention category,

with 71% of these studies resulting in efficacy, compared to 89% in all the other routes combined (Figure 4D), although this difference did not reach statistical significance (P=0.08).

Publication bias significantly overestimated the effect size

Funnel plot demonstrated publication bias (Figure 5). An outlier (lying more than two standard deviations from an otherwise tightly distributed dataset) was noted in the dataset (Figure 5A). To investigate this further, the outlier was disregarded in a *post hoc* analysis and the funnel plot was accordingly replotted (Figure 5B). The recalculated effect size can now be seen to pass directly through the cluster that was previously sitting to the left of the line; thus, the effect size decreases from 5.31 to 3.31. Whilst still statistically significant, this outlier is likely contributing to an overestimation of the estimated effect size of mitochondrial interventions. Furthermore, a structured quality assessment was performed on the included studies; the majority of papers scored highly in certain categories, for example: (i) peer-reviewed articles, (ii) identification of appropriate control groups and (iii) statements regarding conflicts of interest. However, other important categories were not so well-represented. The most strikingly absent criterion was the use of a sample size calculation which, although one of the pivotal aspects of designing a study, was used in only 2 out of 88 papers. Despite this, two-way ANOVA showed that the quality of the research did not significantly impact the efficacy of the interventions being tested (Figure 5C; supplemental table 1).

For Review Only

Discussion

In summary, the data show that there is likely to be potential in targeting mitochondrial dysfunction in ALS (Test for overall effect Z = 5.31, P < 0.00001), with particular strength in interventions prior to disease onset (Test for overall effect Z = 4.66, P < 0.00001). These findings are promising, since they have the potential to form not only the basis of future treatments for ALS, but they also encourage mechanistic study focused on mitochondrial dysfunction, because of the inference that this is likely to be an early, perhaps presymptomatic, pathological event. Before discussing the implications of our findings, it is worth considering the strengths and weaknesses of our approach, and the limitations of the

findings. A major strength of our overview approach is that this is the first explicit systematic review, meta-analysis and structured quality assessment of the preclinical ALS literature examining the influence of interventions targeting mitochondrial dysfunction on survival. Preclinical studies underpin our understanding of disease mechanisms and are crucial for testing interventions for safety and efficacy. However, they come with the caveat that animal studies are inherently heterogeneous – much more so than a clinical trial[13] – and so they are useful only if heterogeneity and their impact on the effect size are taken into consideration. Accordingly, our approach, combining systematic review and meta-analysis, addresses this.

The main weakness stems from the eligibility criteria, by virtue of the fact that studies not reporting alterations in survival were excluded. Thus, following the screening process, 135 studies were included in the meta-analysis. However, only 88 of these studies investigated survival in their models used and, therefore, 47 papers were automatically excluded. It would be desirable to incorporate alternative outcome measures (such as, for example, motor neurone counts, molecular biomarkers, body mass, rotarod performance, gait analysis, and behavioural outcomes) but they need to be fully characterised, quantified and reported in the model. Such outcomes have the potential to translate into surrogates of quality of life outcomes relevant to people with ALS and their caregivers.

Study limitations

First, we show that the overall quality of the studies conducted in the preclinical field of mitochondrial dysfunction in ALS is poor. Using a modification of the CAMARADES criteria[14], an established quality score for assessing overall methodological quality of individual studies in the preclinical literature, a structured quality assessment was performed for each paper included in the meta-analysis. The majority of papers scored highly in certain categories, for example: (i) peer-reviewed articles, (ii) identification of appropriate control groups and (iii) statements regarding conflicts of interest. However, other important categories were not so well-represented. The most strikingly absent criterion was the use of a sample size calculation which, although one of the pivotal aspects of designing a study, was used in only 2 out of 88 papers. Since it is impossible to study a whole population, it is necessary to obtain a fully representative sample using a sample size calculation. Without it,

For Review Only

a sample may be too small to detect a significant result, or too large, with consequent ethical and financial implications. Furthermore, if riluzole, which has been shown to have a limited, but significant, effect in prolonging survival in patients with ALS, is used as a benchmark positive control compound in the most widely published mouse model of ALS, assuming a modest effect size (*circa* 1%), even studies of $n > 50$ animals per cohort would be insufficiently powered to detect it[15]. Given that the sample sizes in the studies included in the meta-analysis ranged from 4 to 39 (median = 13), it is likely that the vast majority, if not all, of these were underpowered. There was also a distinct lack of random allocation and blinding in a large number of the studies, subjecting these and their effect sizes to unknown and unnecessary bias. An important initiative set out with the aim to replicate several studies published in the preclinical domain in ALS found that there was consistent over-estimation of the true effect size[16]. It is therefore possible that the overall effect size calculated in our study may be over-inflated due to widespread overestimation of the effect size studies particularly predating 2010, when guidelines were drawn up[17].

Second, out of the 88 studies that were included in the meta-analysis, 76 used SOD1 G93A transgenic mouse models, where there is overexpression (usually 23 copies) of human genomic mutant *SOD1*[18]. Although this model was and remains important to the understanding of disease mechanisms[19], the over-representation of studies using this model needs to be considered. SOD1 mutation accounts for a minority (<2%) of ALS patients and importantly SOD1 cases do not display the pathological hallmark of ALS, namely TDP-43 proteinopathy, that is found in >97% of ALS cases[20]. Thus, SOD1 transgenic models are likely to recapitulate only certain aspects of ALS. Notwithstanding this, we also encountered difficulties from the lack of reporting the transgene copy number and death from non-ALS causes in the studies incorporated in our review. Both of these variables affect the conclusions that one can reliably draw, given that it is well-known that high copy number models show faster disease progression and earlier death compared with those adopting a low copy mice[21], and these mice may have a heightened susceptibility to infections and other non-ALS related illnesses[16,22].

Finally, whilst there is substantial homology between animal models and humans in terms of conserved genes, organ systems and systemic physiology there are also many important

For Review Only

differences. Indeed the mitochondrial genome, a double-stranded closed-circular molecule, has substantial homology between human and rodent species with 16 569 base pairs in humans and 16 301 base pairs in the mouse[23]. However, there are important functional differences, on an organismal level, in ageing[24] and metabolism[25], including fundamental differences in the production rate of reactive oxygen species between mice and humans[26]. A well-established manifestation of such a difference relates to the influence of calorie restriction on phenotypic measures of mitochondrial function. Several preclinical studies have demonstrated, through caloric restriction, a protective effect on survival through the upregulation of protective mitochondrial pathways associated with apoptosis, reduced reactive oxygen species and an overall reduction in metabolic demand. Indeed, calorie restriction has been shown to extend lifespan in many laboratory organisms and play a preventative role in ageing and diseases including diabetes and cardiovascular diseases[27]. However, a large systematic review and meta-analysis of dietary restriction in the animal model literature concluded that this is more likely to be the result of adaption to laboratory conditions rather than evolutionary conservation[28]. Indeed, evidence for translation of the protective effects of calorie restriction to humans is poor[29]. It is therefore possible that pathways identified through this review may demonstrate efficacy in animal models, but their translation to human therapeutics is limited, highlighting the need for further evaluation in human-based models.

Putative molecular pathways

The most targeted pathway in the extant preclinical literature is the antioxidative stress pathway. However, no pathway has been shown to be better than any others and no intervention has been tested more than three times, questioning the reproducibility of findings. We suggest that this is likely to be in part as a result of the research tools that have been used to detect putative targets (such as the cleaved caspase 3 assay), rather than pure specificity of the target *per se*. Some pathways are under-represented, such as studies examining for defects in the autophagic turnover of damaged mitochondria, termed mitophagy, despite their being recent advances in tools to examine both mitophagy and mitochondrial architecture simultaneously *in vivo*, such as *mito*-QC. There is also a paucity of literature specifically exploring the influence of aberrant mitochondrial pathways in glia *in vivo* including their dynamics[30]. Interestingly there were very few studies adopting

For Review Only

interventions that were specific mitochondrially targeted, for instance, through conjugation to the lipophilic cation, triphenyl phosphonium[31], with the majority of studies adopting interventions that had a cell-wide distribution. The latter is a contributor to the limited success of interventions, despite their promising theoretical mechanism of action, because only a fraction of the compound is taken up by the mitochondria.

Mode of administration of intervention

We show that oral administration was the most frequent mode of intervention and there was a trend towards greater efficacy in studies adopting other routes of administration. This most likely reflects the poor oral bioavailability of oral medications and has been cited as a major flaw in previously conducted clinical trials in the ALS field[32], notwithstanding the difficulties many patients face with swallowing, owing to brainstem dysfunction.

Implications for future research

Through conducting this systematic review and meta-analysis, we conclude that targeting mitochondrial dysfunction in ALS is likely to hold therapeutic potential and that further research should be carried out to delve into the mechanisms underlying the deficit and to further investigate the optimal timing of intervention. We recommend that preclinical research should adopt a wide variety of models[19,33], particularly of mutations accounting for the commoner causes of ALS, such as the *C9orf72* repeat expansion[34,35]. Each model must be fully characterized and suitably powered, and a range of outcome measures reported, in addition to survival, at various time points, particularly later time points, following the onset of symptoms, when interventions are likely to have the greatest translational benefit and where there currently is a paucity of studies. Pharmacological compounds ought to be mitochondrially targeted, with thought given to the suitable route of administration to maximise bioavailability, and coupled to a more comprehensive examination of their effects on multiple mitochondrial signaling and molecular pathways. Finally, technological advances in human induced pluripotent stem cells (iPSCs) and gene editing offer unprecedented opportunities to develop new experimental human models of monogenic neurological diseases[36]. Human iPSCs maximise human relevance, mechanistic insight and also afford high throughput[37]. They uniquely facilitate the study of the non-cell-autonomous influences of mitochondrial gene transcription, owing to the ability to co-

https://mc.manuscriptcentral.com/braincom

For Review Only

culture mixed species-derived neurones with human iPSC-derived astrocytes (for example) and the identification of signaling pathways through *in silico* RNA sequencing read sorting[38]. Human iPSCs also permit the study of live cells in assays examining disease mechanistic vulnerability and sufficiency, and, as such, we recommend that they specifically focus on investigating perturbations in mitochondrial pathways adhering to recent published guidelines[39], expanding the repertoire of ALS disease models.

Acknowledgements and competing interests

A.R.M. is a Lady Edith Wolfson Clinical Fellow and is jointly funded by the Medical Research Council and the Motor Neurone Disease Association. He also acknowledges support from the Rowling Scholars scheme, administered by the Anne Rowling Regenerative Neurology Clinic, University of Edinburgh, Edinburgh, UK. The Chandran laboratory is supported by The Euan MacDonald Centre and the UK Dementia Research Institute partner funders: the Medical Research Council, Alzheimer's Research UK and the Alzheimer's Society.

The authors declare no conflicts of interest.

For Review Only

Data availability statement

All data have been made available through a supplementary data file.

References

1. Brown RH & Al-Chalabi A. (2017). Amyotrophic Lateral Sclerosis. *N Engl J Med* **377,** 162-172.

2. Group W & Group EM-AS. (2017). Safety and efficacy of edaravone in well defined patients with amyotrophic lateral sclerosis: a randomised, double-blind, placebo-controlled trial. *Lancet Neurol* **16,** 505-512.

3. Vandoorne T, De Bock K & Van Den Bosch L. (2018). Energy metabolism in ALS: an underappreciated opportunity? *Acta Neuropathol* **135,** 489-509.

4. Dupuis L, Pradat PF, Ludolph AC & Loeffler JP. (2011). Energy metabolism in amyotrophic lateral sclerosis. *Lancet Neurol* **10,** 75-82.

5. Cozzolino M & Carrì MT. (2012). Mitochondrial dysfunction in ALS. *Prog Neurobiol* **97,** 54-66.

6. Sasaki S & Iwata M. (1996). Ultrastructural study of the synapses of central chromatolytic anterior horn cells in motor neuron disease. *J Neuropathol Exp Neurol* **55,** 932-939.

7. Siklós L, Engelhardt J, Harati Y, Smith RG, Joó F & Appel SH. (1996). Ultrastructural evidence for altered calcium in motor nerve terminals in amyotropic lateral sclerosis. *Ann Neurol* **39,** 203-216.

8. Sasaki S, Horie Y & Iwata M. (2007). Mitochondrial alterations in dorsal root ganglion cells in sporadic amyotrophic lateral sclerosis. *Acta Neuropathol* **114,** 633-639.

9. Zhang F, Wang W, Siedlak SL, Liu Y, Liu J, Jiang K, Perry G, Zhu X & Wang X. (2015). Miro1 deficiency in amyotrophic lateral sclerosis. *Front Aging Neurosci* **7,** 100.

10. Wiedemann FR, Manfredi G, Mawrin C, Beal MF & Schon EA. (2002). Mitochondrial DNA and respiratory chain function in spinal cords of ALS patients. *J Neurochem* **80,** 616-625.

11. Borthwick GM, Johnson MA, Ince PG, Shaw PJ & Turnbull DM. (1999). Mitochondrial enzyme activity in amyotrophic lateral sclerosis: implications for the role of mitochondria in neuronal cell death. *Ann Neurol* **46,** 787-790.

12. Fujimori K, Ishikawa M, Otomo A, Atsuta N, Nakamura R, Akiyama T, Hadano S, Aoki M, Saya H, Sobue G & Okano H. (2018). Modeling sporadic ALS in iPSC-derived motor neurons identifies a potential therapeutic agent. *Nat Med* **24,** 1579-1589.

13. Vesterinen HM, Sena ES, Egan KJ, Hirst TC, Churolov L, Currie GL, Antonic A, Howells DW & Macleod MR. (2014). Meta-analysis of data from animal studies: a practical guide. *J Neurosci Methods* **221,** 92-102.

14. Macleod MR, O'Collins T, Howells DW & Donnan GA. (2004). Pooling of animal experimental data reveals influence of study design and publication bias. *Stroke* **35,** 1203-1208.

15. Scott S, Kranz JE, Cole J, Lincecum JM, Thompson K, Kelly N, Bostrom A, Theodoss J, Al-Nakhala BM, Vieira FG, Ramasubbu J & Heywood JA. (2008). Design, power, and interpretation of studies in the standard murine model of ALS. *Amyotroph Lateral Scler* **9,** 4-15.

16. Perrin S. (2014). Preclinical research: Make mouse studies work. *Nature* **507,** 423-425.

17. Ludolph AC, Bendotti C, Blaugrund E, Chio A, Greensmith L, Loeffler JP, Mead R, Niessen HG, Petri S, Pradat PF, Robberecht W, Ruegg M, Schwalenstöcker B, Stiller D,

For Review Only

van den Berg L, Vieira F & von Horsten S. (2010). Guidelines for preclinical animal research in ALS/MND: A consensus meeting. *Amyotroph Lateral Scler* **11,** 38-45.

18. Gurney ME, Pu H, Chiu AY, Dal Canto MC, Polchow CY, Alexander DD, Caliendo J, Hentati A, Kwon YW & Deng HX. (1994). Motor neuron degeneration in mice that express a human Cu,Zn superoxide dismutase mutation. *Science* **264,** 1772-1775.

19. Van Damme P, Robberecht W & Van Den Bosch L. (2017). Modelling amyotrophic lateral sclerosis: progress and possibilities. *Dis Model Mech* **10,** 537-549.

20. Neumann M, Sampathu DM, Kwong LK, Truax AC, Micsenyi MC, Chou TT, Bruce J, Schuck T, Grossman M, Clark CM, McCluskey LF, Miller BL, Masliah E, Mackenzie IR, Feldman H, Feiden W, Kretzschmar HA, Trojanowski JQ & Lee VM-Y. (2006). Ubiquitinated TDP-43 in Frontotemporal Lobar Degeneration and Amyotrophic Lateral Sclerosis. *Science* **314,** 130-133.

21. Turner BJ & Talbot K. (2008). Transgenics, toxicity and therapeutics in rodent models of mutant SOD1-mediated familial ALS. *Prog Neurobiol* **85,** 94-134.

22. van der Worp HB, Howells DW, Sena ES, Porritt MJ, Rewell S, O'Collins V & Macleod MR. (2010). Can animal models of disease reliably inform human studies? *PLoS Med* **7,** e1000245.

23. Wallace DC & Fan W. (2009). The pathophysiology of mitochondrial disease as modeled in the mouse. *Genes Dev* **23,** 1714-1736.

24. Demetrius L. (2006). Aging in mouse and human systems: a comparative study. *Ann N Y Acad Sci* **1067,** 66-82.

25. Svenson KL, Von Smith R, Magnani PA, Suetin HR, Paigen B, Naggert JK, Li R, Churchill GA & Peters LL. (2007). Multiple trait measurements in 43 inbred mouse strains capture the phenotypic diversity characteristic of human populations. *J Appl Physiol (1985)* **102,** 2369-2378.

26. Finkel T & Holbrook NJ. (2000). Oxidants, oxidative stress and the biology of ageing. *Nature* **408,** 239-247.

27. Partridge L, Brand, M.D. (2005). Special issue on dietary restriction: Dietary restriction, longevity and ageing—the current state of our knowledge and ignorance. *Mechanisms of Ageing and Development* **126,** 911 - 912.

28. Nakagawa S, Lagisz M, Hector KL & Spencer HG. (2012). Comparative and meta-analytic insights into life extension via dietary restriction. *Aging Cell* **11,** 401-409.

29. Phelan JP & Rose MR. (2005). Why dietary restriction substantially increases longevity in animal models but won't in humans. *Ageing Res Rev* **4,** 339-350.

For Review Only

30. Jackson JG & Robinson MB. (2018). Regulation of mitochondrial dynamics in astrocytes: Mechanisms, consequences, and unknowns. *Glia* **66,** 1213-1234.

31. Zielonka J, Joseph J, Sikora A, Hardy M, Ouari O, Vasquez-Vivar J, Cheng G, Lopez M & Kalyanaraman B. (2017). Mitochondria-Targeted Triphenylphosphonium-Based Compounds: Syntheses, Mechanisms of Action, and Therapeutic and Diagnostic Applications. *Chem Rev* **117,** 10043-10120.

32. Mitsumoto H, Brooks BR & Silani V. (2014). Clinical trials in amyotrophic lateral sclerosis: why so many negative trials and how can trials be improved? *Lancet Neurol* **13,** 1127-1138.

33. Lutz C. (2018). Mouse models of ALS: Past, present and future. *Brain Res* **1693,** 1-10.

34. DeJesus-Hernandez M, Mackenzie IR, Boeve BF, Boxer AL, Baker M, Rutherford NJ, Nicholson AM, Finch NA, Flynn H, Adamson J, Kouri N, Wojtas A, Sengdy P, Hsiung GY, Karydas A, Seeley WW, Josephs KA, Coppola G, Geschwind DH, Wszolek ZK, Feldman H, Knopman DS, Petersen RC, Miller BL, Dickson DW, Boylan KB, Graff-Radford NR & Rademakers R. (2011). Expanded GGGGCC hexanucleotide repeat in noncoding region of C9ORF72 causes chromosome 9p-linked FTD and ALS. *Neuron* **72,** 245-256.

35. Renton AE, Majounie E, Waite A, Simón-Sánchez J, Rollinson S, Gibbs JR, Schymick JC, Laaksovirta H, van Swieten JC, Myllykangas L, Kalimo H, Paetau A, Abramzon Y, Remes AM, Kaganovich A, Scholz SW, Duckworth J, Ding J, Harmer DW, Hernandez DG, Johnson JO, Mok K, Ryten M, Trabzuni D, Guerreiro RJ, Orrell RW, Neal J, Murray A, Pearson J, Jansen IE, Sondervan D, Seelaar H, Blake D, Young K, Halliwell N, Callister JB, Toulson G, Richardson A, Gerhard A, Snowden J, Mann D, Neary D, Nalls MA, Peuralinna T, Jansson L, Isoviita VM, Kaivorinne AL, Hölttä-Vuori M, Ikonen E, Sulkava R, Benatar M, Wuu J, Chiò A, Restagno G, Borghero G, Sabatelli M, Heckerman D, Rogaeva E, Zinman L, Rothstein JD, Sendtner M, Drepper C, Eichler EE, Alkan C, Abdullaev Z, Pack SD, Dutra A, Pak E, Hardy J, Singleton A, Williams NM, Heutink P, Pickering-Brown S, Morris HR, Tienari PJ, Traynor BJ & Consortium I. (2011). A hexanucleotide repeat expansion in C9ORF72 is the cause of chromosome 9p21-linked ALS-FTD. *Neuron* **72,** 257-268.

36. Sandoe J & Eggan K. (2013). Opportunities and challenges of pluripotent stem cell neurodegenerative disease models. *Nat Neurosci* **16,** 780-789.

37. Dolmetsch R & Geschwind DH. (2011). The human brain in a dish: the promise of iPSC-derived neurons. *Cell* **145,** 831-834.

38. Qiu J, Dando O, Baxter PS, Hasel P, Heron S, Simpson TI & Hardingham GE. (2018). Mixed-species RNA-seq for elucidation of non-cell-autonomous control of gene transcription. *Nat Protoc* **13,** 2176-2199.

39. Connolly NMC, Theurey P, Adam-Vizi V, Bazan NG, Bernardi P, Bolaños JP, Culmsee C, Dawson VL, Deshmukh M, Duchen MR, Düssmann H, Fiskum G, Galindo MF,

For Review Only

Hardingham GE, Hardwick JM, Jekabsons MB, Jonas EA, Jordán J, Lipton SA, Manfredi G, Mattson MP, McLaughlin B, Methner A, Murphy AN, Murphy MP, Nicholls DG, Polster BM, Pozzan T, Rizzuto R, Satrústegui J, Slack RS, Swanson RA, Swerdlow RH, Will Y, Ying Z, Joselin A, Gioran A, Moreira Pinho C, Watters O, Salvucci M, Llorente-Folch I, Park DS, Bano D, Ankarcrona M, Pizzo P & Prehn JHM. (2018). Guidelines on experimental methods to assess mitochondrial dysfunction in cellular models of neurodegenerative diseases. *Cell Death Differ* **25,** 542-572.

Figure legends

Figure 1: PRISMA flowchart

A. PRISMA flowchart indicating numbers of studies at each stage of the review. *Studies were excluded based on the following reasons: (i) due to inappropriate data formatting (*i.e.,* where no useable data could be extracted); (ii) conference poster abstract with insufficient data; or (iii) inappropriately screened (*i.e.,* where the study did not meet inclusion/exclusion criteria). For full dataset see supplementary table 1.

For Review Only

Figure 2: Meta-analysis of 88 preclinical studies shows therapeutic potential for targeting mitochondrial pathways in ALS

Forest plot showing the odds ratio and confidence intervals calculated from survival summary data from each study, weighted by study size. Overall effect estimate is demonstrated (with 95% confidence intervals) as a black diamond at the bottom of the graph. Heterogeneity is displayed as an I^2 value. Results demonstrate an overall statistically significant effect favouring targeting of mitochondrial pathways in ALS.

Figure 3: The majority of studies are conducted at early time points and show that with early interventions there is a statistically significant improvement in survival

Forest plots showing the odds ratio and confidence intervals calculated from survival summary data from each study, weighted by study size. Overall effect estimate is demonstrated (with 95% confidence intervals) as a black diamond at the bottom of the graph. Heterogeneity is displayed as an I^2 value. **A.** Interventions delivered pre-symptom onset; **B.** at symptom onset and **C.** after symptom onset. **D**. Stacked frequency histogram illustrating the number of studies demonstrating efficacy (blue) as a proportion of the total number of studies (remaining white bar), divided into categories depending on timing of administered intervention. Results demonstrate an overall statistically significant effect favouring the targeting of mitochondrial pathways early in ALS (A); however, there is no difference in likelihood of demonstrating efficacy depending on timing of intervention (D), implying that there are too few studies conducted at later time points (2-way ANOVA, $P>0.05$).

Figure 4: Subgroup analyses reveal no difference in efficacy between divergent pathways, putative target or mode of delivery of intervention

A. Frequency of studies implementing therapeutic interventions that target the following pathways: AO – anti-oxidative stress; AA – anti-apoptosis; MT – metabolism; AI – anti-inflammation; MD – mitophagy/ degradation;

ETC – electron transport chain; CB – calcium buffering; and EX – excitotoxicity. Each column shows studies demonstrating efficacy in blue and no efficacy in white. 2-way ANOVA shows no statistically significant difference (P>0.05). **B.** Frequency of studies implementing interventions that specifically target mitochondria (MT) *vs* more generic cell-wide distribution (CWD). Each column shows studies demonstrating efficacy in blue and no efficacy in white. Chi^2 demonstrates no statistically significant difference (P=0.14). **C.** Frequency of the mode of delivery of study interventions: PO – pharmaceutical oral; PIP – pharmaceutical intraperitoneal; PSC – pharmaceutical subcutaneous; PIV – pharmaceutical intravenous; CTS – cell transplant specific cell-type indicated; CTU – cell transplant no specific cell type indicated; PCNS – pharmaceutical directly administered to central nervous system; GA – genetic intervention, all cells targeted; U – unknown/not stated. Each column shows studies demonstrating efficacy in blue and no efficacy in white. 2-way ANOVA shows no statistically significant difference (P>0.05). **D.** *Post hoc* analysis performed to compare pharmaceutical oral (PO) *vs* all other modes of delivery grouped together. Chi^2 demonstrates no statistically significant difference (P=0.08).

Figure 5: Publication bias results in overestimation of effect size

A. Funnel plot showing each point as a study, plotted against the effect size of that study (abscissa) and precision of that study (SE(log[OR]); ordinate). One study outlier was identified (highlighted by a blue square), skewing the effect size significantly, resulting in an overestimation of the effect size. **B.** Adjusted funnel plot demonstrating recalculated effect size estimate of 3.31, *cf*. 5.31. **C.** Frequency distribution illustrating that the structured quality score is not significant in determining intervention efficacy (2-way ANOVA P>0.05).

Supplementary table 1:

Table summarising all data extracted from the 88 studies included in the quantitative meta-analysis.

Supplemental file: PRISMA checklist 2009.

For Review Only

Figure 1

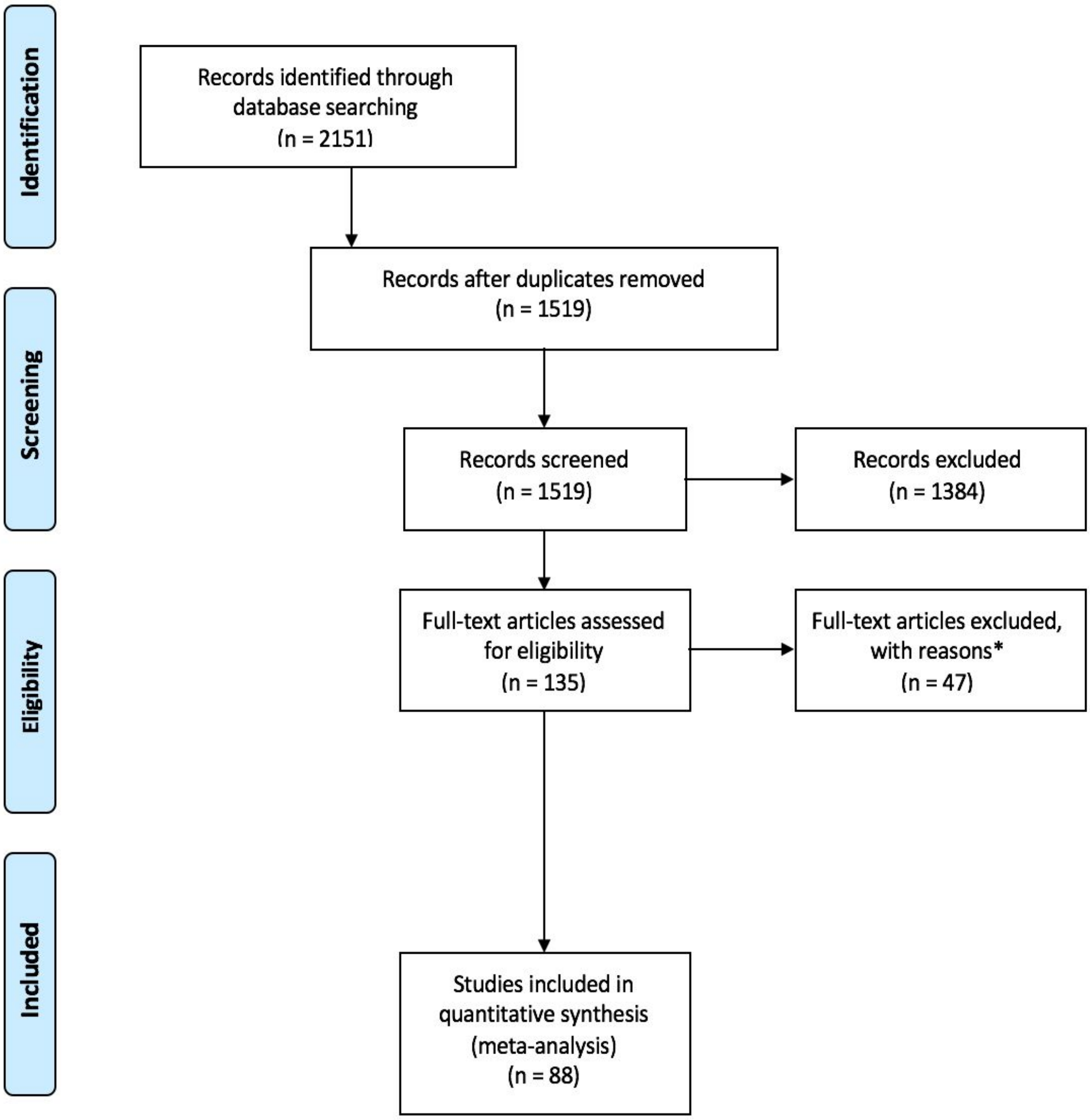
Identification
Screening
Eligibility
Included
Records identified through database searching (n = 2151)
Records after duplicates removed (n = 1519)
Records screened (n = 1519)
Records excluded (n = 1384)
Full-text articles assessed for eligibility (n = 135)
Full-text articles excluded, with reasons* (n = 47)
Studies included in quantitative synthesis (meta-analysis) (n = 88)

Figure 2

Study or Subgroup	log[Odds Ratio]	SE	Weight	Odds Ratio IV, Random, 95% CI
Andries 2007	0.0789474	0.43	0.3%	1.08 [0.47, 2.51]
Apolloni 2014	0.035503	0.2	1.4%	1.04 [0.70, 1.53]
Apolloni 2016	0.0409357	0.28	0.7%	1.04 [0.60, 1.80]
Apolloni 2017	0.0030581	0.24	1.0%	1.00 [0.63, 1.61]
Ari 2014	0.0625	0.25	0.9%	1.06 [0.65, 1.74]
Audet 2011	0	0.29	0.7%	1.00 [0.57, 1.77]
Bartlett 2017	0.013986	0.42	0.3%	1.01 [0.45, 2.31]
Becker 2017	0.28125	0.33	0.5%	1.32 [0.69, 2.53]
Bordet 2007	0.0888889	0.22	1.1%	1.09 [0.71, 1.68]
Bruce 2004	0.1119403	0.1	5.6%	1.12 [0.92, 1.36]
Cacabelos 2014 (1)	0	0.41	0.3%	1.00 [0.45, 2.23]
Cacabelos 2014 (2)	0	0.34	0.5%	1.00 [0.51, 1.95]
Coughlan 2015	0.05325	0.46	0.3%	1.05 [0.43, 2.60]
Coughlan 2016	0.310345	0.29	0.7%	1.36 [0.77, 2.41]
Crow 2005	0.3175676	0.22	1.1%	1.37 [0.89, 2.11]
Danzeisen 2006	0.0526416	0.26	0.8%	1.05 [0.63, 1.75]
Fontanilla 2012	0.0948905	0.72	0.1%	1.10 [0.27, 4.51]
Fornai 2008	0.2361111	0.4	0.3%	1.27 [0.58, 2.77]
Fujisawa 2016	0.0729927	0.36	0.4%	1.08 [0.53, 2.18]
Fujisawa 2016 (2)	0.06273063	0.4	0.3%	1.06 [0.49, 2.33]
Gill 2009	−0.0325203	0.55	0.2%	0.97 [0.33, 2.84]
Goursaud 2015	0.2201835	0.2	1.4%	1.25 [0.84, 1.84]
Groenevald 2003	−0.0703125	0.22	1.1%	0.93 [0.61, 1.43]
Gubert 2016 (1)	0.0230769	0.46	0.3%	1.02 [0.42, 2.52]
Gubert 2016 (2)	0.0137931	0.44	0.3%	1.01 [0.43, 2.40]
Hseuh 2016	0.381188	0.16	2.2%	1.46 [1.07, 2.00]
Hwee 2014	0.0254237	1.62	0.0%	1.03 [0.04, 24.55]
Jiang 2000	0.090909	0.25	0.9%	1.10 [0.67, 1.79]
Jiang 2014	0.0958904	0.3	0.6%	1.10 [0.61, 1.98]
Jin 2007	0.1448276	0.26	0.8%	1.16 [0.69, 1.92]
Kaneb 2011 (1)	0.0390625	0.28	0.7%	1.04 [0.60, 1.80]
Kaneb 2011 (2)	−0.0294118	0.28	0.7%	0.97 [0.56, 1.68]
Katsumata 2012	0.049296	0.56	0.2%	1.05 [0.35, 3.15]
Kiaei 2005	0.125	0.2	1.4%	1.13 [0.77, 1.68]
Kiaei 2005 (2)	0.1111111	0.33	0.5%	1.12 [0.59, 2.13]
Kilvenyi 1999	0.1666667	0.13	3.3%	1.18 [0.92, 1.52]
Kim 2006	0.037313	0.18	1.7%	1.04 [0.73, 1.48]
Kim 2006 (2)	0.00813008	0.14	2.8%	1.01 [0.77, 1.33]
Kim 2014	0.1621622	0.18	1.7%	1.18 [0.83, 1.67]
Kunis 2015	0.0759494	0.42	0.3%	1.08 [0.47, 2.46]
Lange 1983	0	0.1	5.6%	1.00 [0.82, 1.22]
Lee 2017	0.06358382	0.26	0.8%	1.07 [0.64, 1.77]
Lee 2017 (3)	0.06111111	0.22	1.1%	1.06 [0.69, 1.64]
Li 2011	−0.1775701	0.2	1.4%	0.84 [0.57, 1.24]
Li 2015	0.0763359	0.6	0.2%	1.08 [0.33, 3.50]
Llewellyn 2014	0.8888889	0.1	5.6%	2.43 [2.00, 2.96]
Mancuso 2014	0.0945946	0.23	1.1%	1.10 [0.70, 1.73]
Martin 2014	0.4202899	0.22	1.1%	1.52 [0.99, 2.34]
Matsuo 2013	0.1213873	0.3	0.6%	1.13 [0.63, 2.03]
Miquel 2012 (1)	0.0441176	0.18	1.7%	1.05 [0.73, 1.49]
Miquel 2012 (2)	0.1079137	0.18	1.7%	1.11 [0.78, 1.59]
Miquel 2014	0.0530303	0.3	0.6%	1.05 [0.59, 1.90]
Morita 2008	0	0.28	0.7%	1.00 [0.58, 1.73]
Morita 2008 (2)	0.0683223	0.29	0.7%	1.07 [0.61, 1.89]
Neymotin 2009	0.0909091	0.36	0.4%	1.10 [0.54, 2.22]
Neymotin 2011 (1)	0.1388889	0.36	0.4%	1.15 [0.57, 2.33]
Neymotin 2011 (2)	0.1071429	0.36	0.4%	1.11 [0.55, 2.25]
Neymotin 2011 (3)	0.1180556	0.36	0.4%	1.13 [0.56, 2.28]
Neymotin 2011 (4)	0.1197183	0.36	0.4%	1.13 [0.56, 2.28]
Nicaise 2008	0.120332	0.19	1.5%	1.13 [0.78, 1.64]
Ohta 2009	0.0769231	0.28	0.7%	1.08 [0.62, 1.87]
Patel 2010 (1)	−0.1016949	0.17	1.9%	0.90 [0.65, 1.26]
Patel 2010 (2)	−0.1166667	0.37	0.4%	0.89 [0.43, 1.84]
Perera 2017	−0.0129032	0.2	1.4%	0.99 [0.67, 1.46]
Reinholz 1999	0.0416667	0.31	0.6%	1.04 [0.57, 1.91]
Riancho 2015	0.0507246	0.27	0.8%	1.05 [0.62, 1.79]
Ryu 2005	0.125	0.4	0.3%	1.13 [0.52, 2.48]
Saenger 2012	0.0804954	0.12	3.9%	1.08 [0.86, 1.37]
Sagot 2001	0.34375	0.34	0.5%	1.41 [0.72, 2.75]
Solomon 2011	0.0234375	0.34	0.5%	1.02 [0.53, 1.99]
Song 2014	0.1449275	0.2	1.4%	1.16 [0.78, 1.71]
Su 2016	−0.0833333	0.2	1.4%	0.92 [0.62, 1.36]
Takata 2013	0.0615385	0.27	0.8%	1.06 [0.63, 1.81]
Tanaka 2011	−0.1	0.69	0.1%	0.90 [0.23, 3.50]
Turner 2003	0.0503597	0.16	2.2%	1.05 [0.77, 1.44]
Wang 2005	0.0687023	0.21	1.3%	1.07 [0.71, 1.62]
Wang 2007	0.1086957	0.41	0.3%	1.11 [0.50, 2.49]
Wang 2011	0.0935252	0.16	2.2%	1.10 [0.80, 1.50]
Wang 2011 (2)	0.07352941	0.16	2.2%	1.08 [0.79, 1.47]
Wu 2003	0.0447761	0.38	0.4%	1.05 [0.50, 2.20]
Zhang 2009	0.1082803	0.33	0.5%	1.11 [0.58, 2.13]
Zhang 2013	0.0486111	0.3	0.6%	1.05 [0.58, 1.89]
Zhang 2014	0.1448276	0.18	1.7%	1.16 [0.81, 1.64]
Zhang 2016	0.0689655	0.3	0.6%	1.07 [0.60, 1.93]
Zhao 2006	0.045872	0.11	4.6%	1.05 [0.84, 1.30]
Zhao 2012	0.0444444	0.22	1.1%	1.05 [0.68, 1.61]
Zhou 2017	0.1733333	0.4	0.3%	1.19 [0.54, 2.60]
Zhu 2002	0.0661765	0.16	2.2%	1.07 [0.78, 1.46]
**Total (95% CI)**			**100.0%**	**1.13 [1.08, 1.19]**

Heterogeneity: $Tau^2 = 0.00$; $Chi^2 = 78.94$, df = 87 (P = 0.72); $I^2 = 0\%$
Test for overall effect: Z = 5.31 (P < 0.00001)

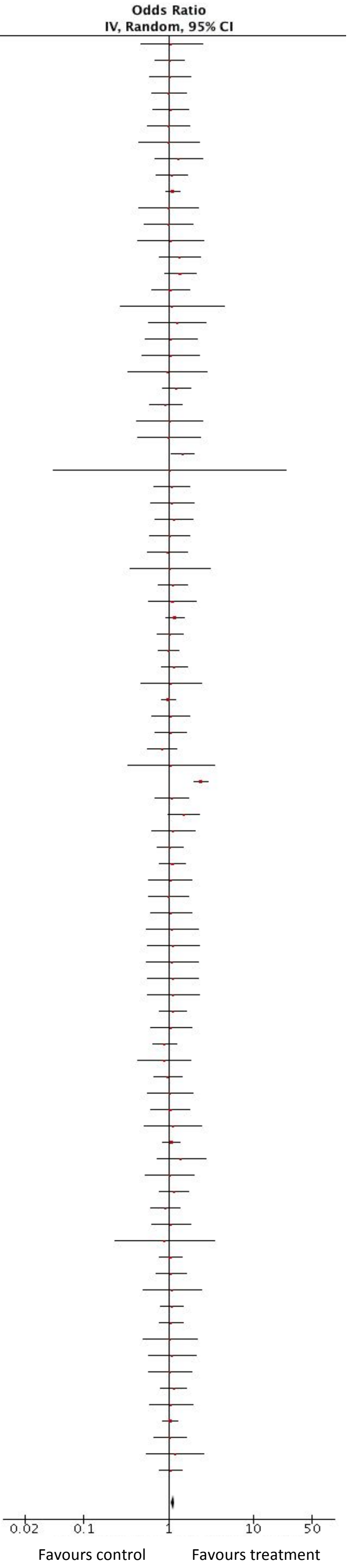

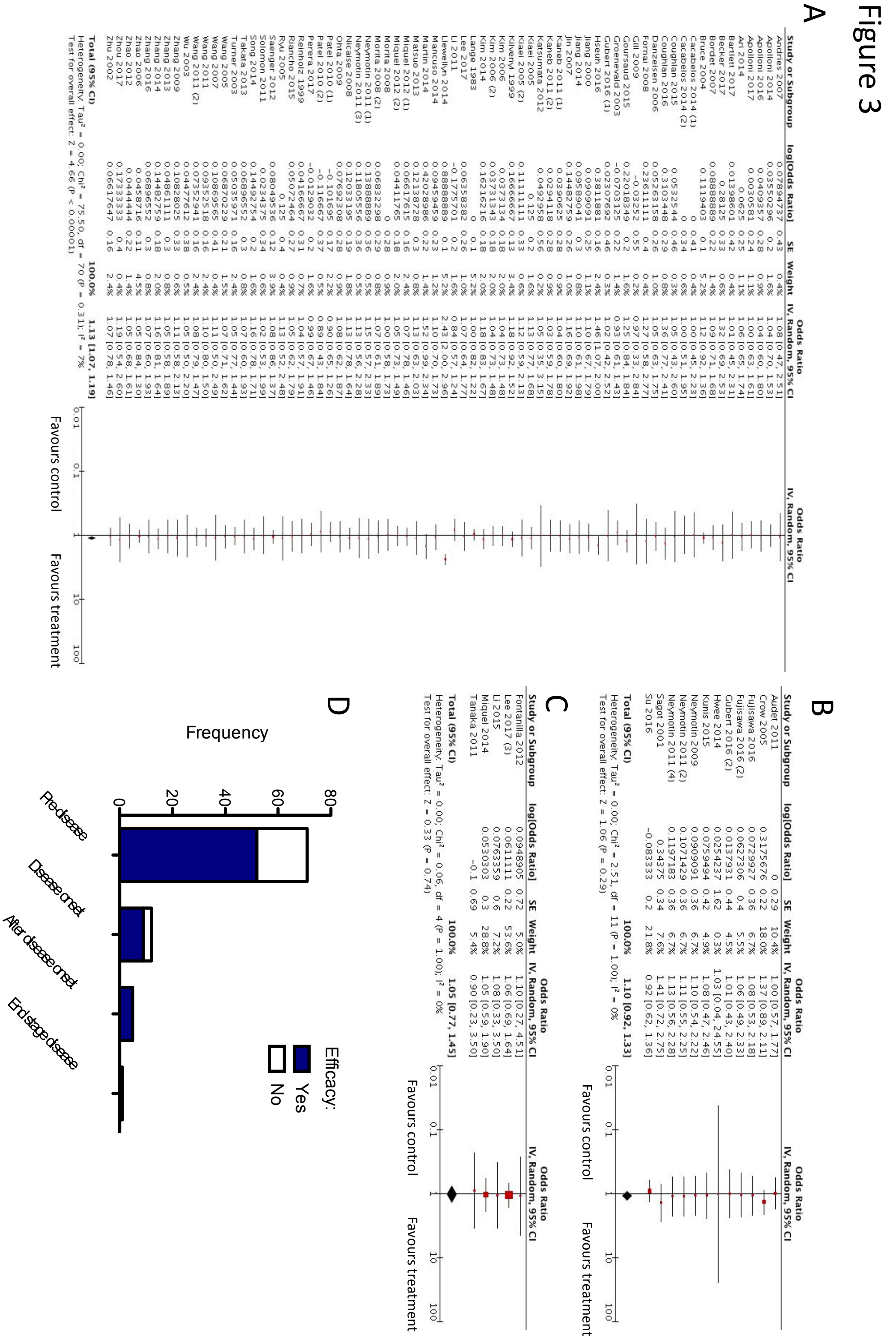
Figure 3
A
B
C
D
Study or Subgroup
log[Odds Ratio]
SE
Weight
Odds Ratio IV, Random, 95% CI
Favours control
Favours treatment
Frequency
Pre disease
Disease onset
After disease onset
End stage disease
Efficacy:
Yes
No

Figure 4

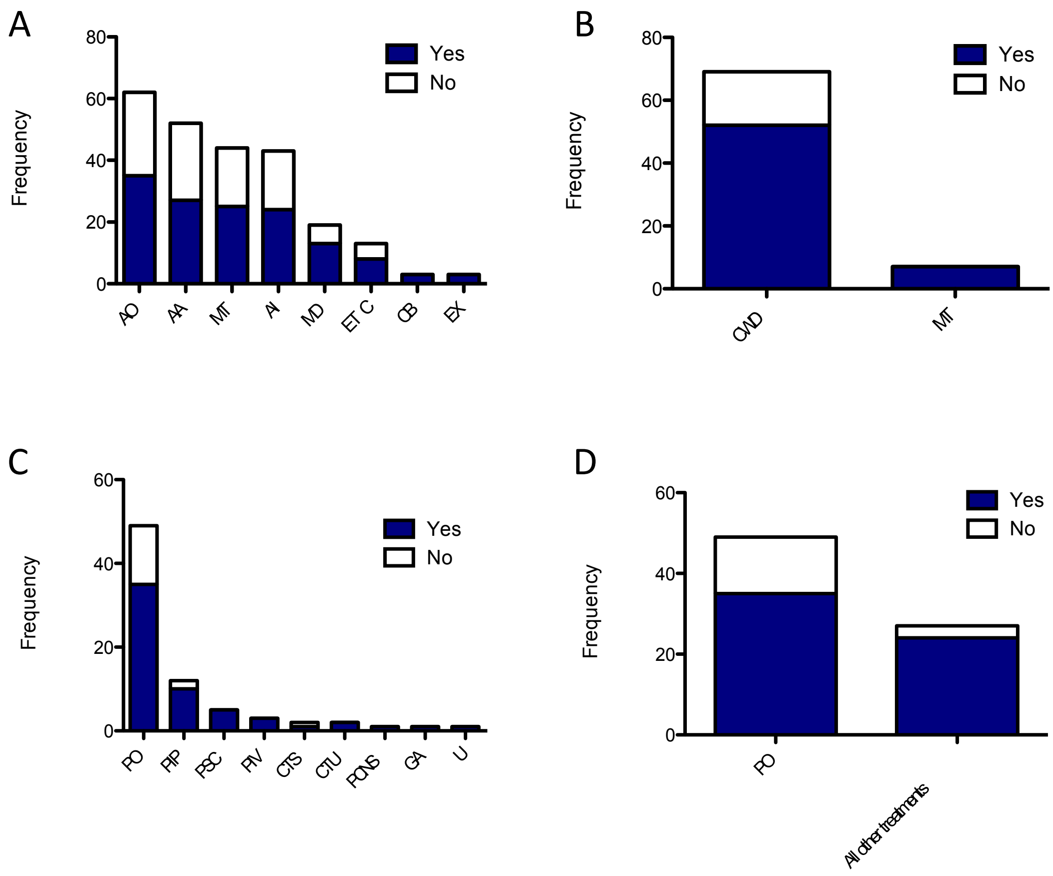
A
B
C
D
Frequency
Yes
No
AO
AA
MT
AI
MD
ET C
CB
EX
OWD
PO
PIP
PSC
PIV
CTS
CTU
PONS
GA
U
All other treatments

Figure 5

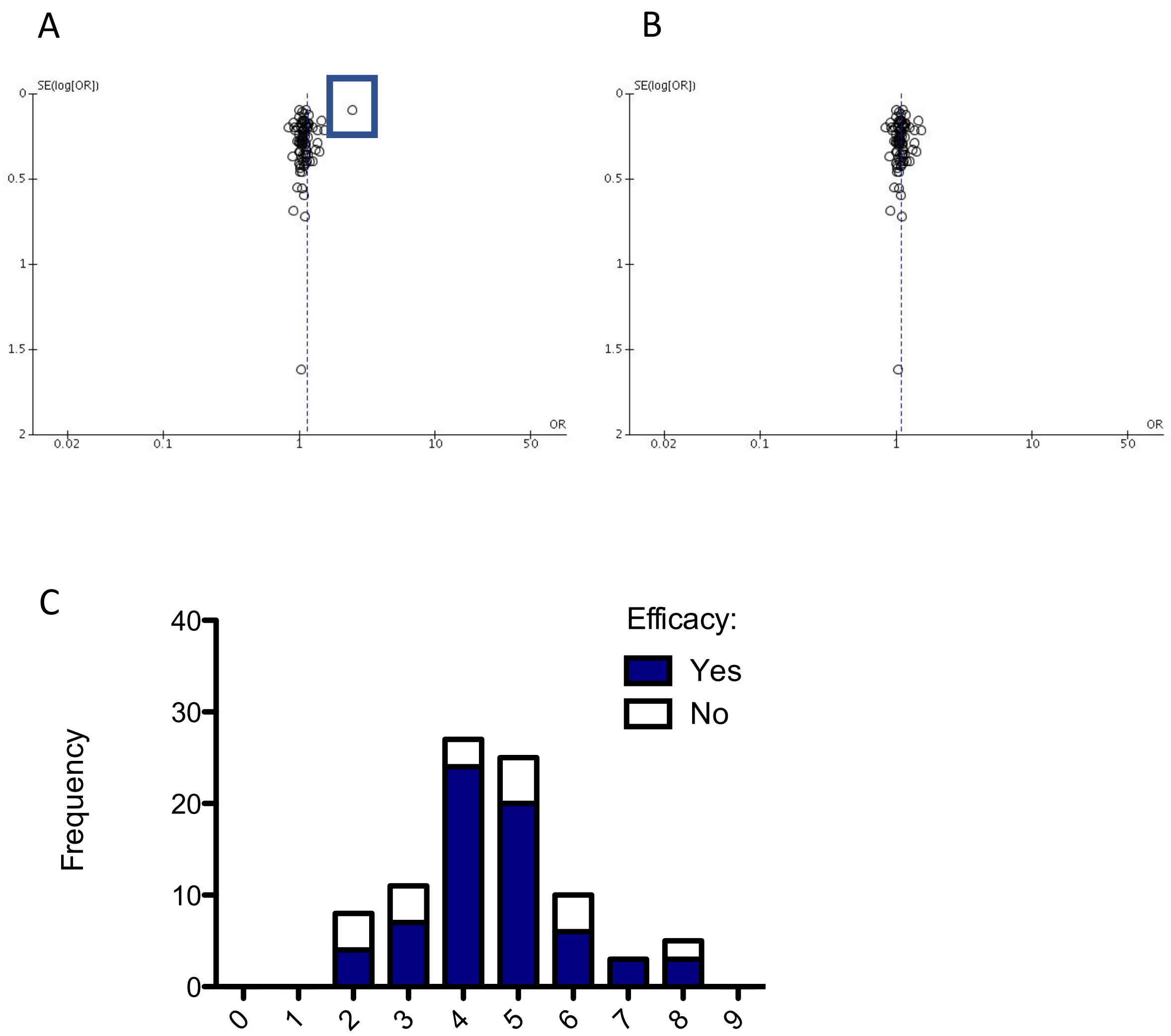
A
SE(log[OR])
0
0.5
1
1.5
2
0.02
0.1
1
10
50
OR
B
SE(log[OR])
0
0.5
1
1.5
2
0.02
0.1
1
10
50
OR
C
Frequency
40
30
20
10
0
0
1
2
3
4
5
6
7
8
9
Efficacy:
Yes
No

**Author**
K. S. Coughlan
K. S. Coughlan
Z. Zhao
E. Morita
D. J. Lange
K. Kim
Y. Sagot
K. W. Hsueh
F. Jiang
K. Tanaka
R. Katsumata
C. V. Fontanilla
B. P. Patel
W. Zhao
M. Kiaei
K. M. Bruce
A. Gill
S. Apolloni
H. S. Jin
D. Cacabelos
Y. Zhang
D. T. Hwee
M. Takata
S. Saenger
C. Nicaise
T. Matsuo
L. J. Martin
H. Q. Jiang
T. Bordet
G. Kunis
F. Gubert
M. Keep
A. S. Wu
M. Andries
A. Neymotin
K. J. Llewellyn
F. Fornai
J. P. Crow

For Review Only

Y. Zhang
R. Wang
C. Ari
H. M. Kaneb
J. N. Audet
S. Zhu
E. Miquel
X. Zhang
C. Zhang
W. Li
Q. M. Zhou
J. Riancho
A. Neymotin
P. Klivenyi
E. Miquel
H. Wang
B. J. Turner
R. Bartlett
S. Apolloni
M. Kiaei
J. D. Lee
Q. Wang
L. Li
L. Song
R. Mancuso
N. D. Perera
H. Ryu
S. Apolloni
X. W. Su
R. Danzeisen
S. Goursaud
T. Fujisawa
G. J. Groeneveld
Y. Ohta
M. M. Reinholz
J. A. Solomon
L. A. Becker
K. S. Kim

For Review Only

Summary stats

For Review Only

Title
Preconditioning with latrepirdine, an adenosine 5'-monophosphate-activated protein kinase activat
A high-fat jelly diet restores bioenergetic balance and extends lifespan in the presence of motor dys
A ketogenic diet as a potential novel therapeutic intervention in amyotrophic lateral sclerosis
A novel cell transplantation protocol and its application to an ALS mouse model
A therapeutic trial of gangliosides and thymosin in the Wobbler mouse model of motor neuron dise
AM1241, a cannabinoid CB2 receptor selective compound, delays disease progression in a mouse m
An orally active anti-apoptotic molecule (CGP 3466B) preserves mitochondria and enhances surviva
Autophagic down-regulation in motor neurons remarkably prolongs the survival of ALS mice
Beneficial effect of ginseng root in SOD-1 (G93A) transgenic mice
Bromocriptine methylate suppresses glial inflammation and moderates disease progression in a mo
c-Abl inhibition delays motor neuron degeneration in the G93A mouse, an animal model of amyotro
Caffeic acid phenethyl ester extends survival of a mouse model of amyotrophic lateral sclerosis
Caloric restriction shortens lifespan through an increase in lipid peroxidation, inflammation and apo
Caprylic triglyceride as a novel therapeutic approach to effectively improve the performance and at
Celastrol blocks neuronal cell death and extends life in transgenic mouse model of amyotrophic late
Chemotherapy delays progression of motor neuron disease in the SOD1 G93A transgenic mouse
Chronic lithium dosing in a sibling-matched, gender balanced, investigator-blinded trial using a stan
Clemastine Confers Neuroprotection and Induces an Anti-Inflammatory Phenotype in SOD1(G93A)
Concurrent administration of Neu2000 and lithium produces marked improvement of motor neuro
Dietary Lipid Unsaturation Influences Survival and Oxidative Modifications of an Amyotrophic Later
Treatment with Hydrogen-Rich Saline Delays Disease Progression in a Mouse Model of Amyotrophi
Fast skeletal muscle troponin activator tirasemtiv increases muscle function and performance in the
Fasudil, a rho kinase inhibitor, limits motor neuron loss in experimental models of amyotrophic late
Functional improvement in mouse models of familial amyotrophic lateral sclerosis by PEGylated ins
Gemals, a new drug candidate, extends lifespan and improves electromyographic parameters in a r
Ghrelin attenuates disease progression in a mouse model of amyotrophic lateral sclerosis
GNX-4728, a novel small molecule drug inhibitor of mitochondrial permeability transition, is therap
Guanabenz delays the onset of disease symptoms, extends lifespan, improves motor performance a
Identification and characterization of cholest-4-en-3-one, oxime (TRO19622), a novel drug candidat
Immunization with a myelin-derived antigen activates the brain's choroid plexus for recruitment of
Intraspinal bone-marrow cell therapy at pre- and symptomatic phases in a mouse model of amyotro
Intrathecal cyclosporin prolongs survival of late-stage ALS mice
Iron porphyrin treatment extends survival in a transgenic animal model of amyotrophic lateral scler
Ivermectin inhibits AMPA receptor-mediated excitotoxicity in cultured motor neurons and extends
Lenalidomide (Revlimid) administration at symptom onset is neuroprotective in a mouse model of a
Lipid-enriched diet rescues lethality and slows down progression in a murine model of VCP-associat
Lithium delays progression of amyotrophic lateral sclerosis
Manganese porphyrin given at symptom onset markedly extends survival of ALS mice

For Review Only

Melatonin inhibits the caspase-1/cytochrome c/caspase-3 cell death pathway, inhibits MT1 recepto
Memantine prolongs survival in an amyotrophic lateral sclerosis mouse model
Metabolic therapy with Deanna Protocol supplementation delays disease progression and extends
Metformin treatment has no beneficial effect in a dose-response survival study in the SOD1 G93A n
Methylene blue administration fails to confer neuroprotection in two amyotrophic lateral sclerosis
Minocycline inhibits cytochrome c release and delays progression of amyotrophic lateral sclerosis ir
Modulation of astrocytic mitochondrial function by dichloroacetate improves survival and motor pe
MTOR-independent, autophagic enhancer trehalose prolongs motor neuron survival and ameliorat
Multiple administrations of human marrow stromal cells through cerebrospinal fluid prolong surviv
N-acetyl-L-tryptophan delays disease onset and extends survival in an amyotrophic lateral sclerosis
n-butylidenephthalide treatment prolongs life span and attenuates motor neuron loss in SOD1 G93
Neuroprotective effect of bexarotene in the SOD1G93A mouse model of amyotrophic lateral sclero
Neuroprotective effect of Nrf2/ARE activators, CDDO ethylamide and CDDO trifluoroethylamide, in
Neuroprotective effects of creatine in a transgenic animal model of amyotrophic lateral sclerosis
Neuroprotective effects of the mitochondria-targeted antioxidant MitoQ in a model of inherited am
Nortriptyline delays disease onset in models of chronic neurodegeneration
Opposing effects of low and high-dose clozapine on survival of transgenic amyotrophic lateral scler
P2X7 antagonism using brilliant blue G reduces body weight loss and prolongs survival in female SO
P2X7 receptor activation modulates inflammation and autophagy in amyotrophic lateral sclerosis m
Peroxisome proliferator-activated receptor-gamma agonist extends survival in transgenic mouse m
Pharmacological inhibition of complement C5a-C5a1 receptor signalling ameliorates disease pathol
Prevention of motor neuron degeneration by novel iron chelators in SOD1 G93A transgenic mice of
Rapamycin treatment augments motor neuron degeneration in SOD1 G93A mouse model of amyot
Resveratrol ameliorates motor neuron degeneration and improves survival in SOD1 G93A mouse m
Resveratrol improves motoneuron function and extends survival in SOD1(G93A) ALS mice
Rilmenidine promotes MTOR-independent autophagy in the mutant SOD1 mouse model of amyotr
Sodium phenylbutyrate prolongs survival and regulates expression of anti-apoptotic genes in transg
Spinal cord pathology is ameliorated by P2X7 antagonism in a SOD1-mutant mouse model of amyot
Statins accelerate disease progression and shorten survival in SOD1(G93A) mice
Targeted antioxidative and neuroprotective properties of the dopamine agonist pramipexole and it
The anti-inflammatory peptide stearyl-norleucine-VIP delays disease onset and extends survival in a
The ASK1-specific inhibitors K811 and K812 prolong survival in a mouse model of amyotrophic later
Zinc amplifies mSOD1-mediated toxicity in a transgenic mouse model of amyotrophic lateral scleros
Therapeutic benefits of intrathecal protein therapy in a mouse model of amyotrophic lateral scleros
Therapeutic benefits of putrescine-modified catalase in a transgenic mouse model of familial amyot
Vitamin D3 deficiency differentially affects functional and disease outcomes in the G93A mouse mo
Therapeutic reduction of ataxin-2 extends lifespan and reduces pathology in TDP-43 mice
Transplantation of human adipose tissue-derived stem cells delays clinical onset and prolongs life s

For Review Only

For Review Only

Year	Survival		
	N = Rx group	Survival in Rx group	N = Control group
2015	24	169	22
2016	15	145	14
2006	6	109	5
2008	17	168	11
	18	161	11
1983	5	140	5
2006	9	134	9
	7	123	7
2001	12	64	22
2016	8	202	8
2000	12	143	13
2011	38	150	31
2012	28	142	28
2012	39	137	33
2010	10	118	7
	21	120	16
2012	11	135	11
2005	10	144	10
2004	6	134	4
2009	28	123	27
2016	16	171	12
2007	13	145	13
2014	28	133	13
	22	127	12
2016	15	145	15
2014	81	118	81
2013	12	130	15
2012	6	323	6
2008	11	241	8
2013	15	173	15
2014	12	690	10
2014	15	146	15
2007	11	135	11
2015	19	158	23
2016	22	130	24
	22	145	22
2001	7	139	5
2003	19	134	19
2007	11	152	32
2009	18	143	18
2014	5	63	5
2008	20	144	20
2005	11	148	11

For Review Only

2013	15	144	15
2005	10	131	11
2014	12	128	13
2011	14	128	14
	14	136	14
2011	10	141	19
2002	8	136	8
2012	9	136	9
	9	139	9
2014	6	145	12
2009	17	157	16
2015	30	131	30
2017	20	150	20
2015	15	138	12
2011	18	144	18
	18	140	18
	18	144	18
	18	142	18
1999	7	168	6
2014	15	132	15
2007	12	138	29
2003	8	139	8
2017	21	143	21
2017	12	163.5	12
2005	18	135	15
2017	11	180	11
	13	173	13
2011	8	136	8
	8	139	8
2011	10	107	10
2014	10	138	10
2014	13	148	10
2017	10	155	10
2005	20	144	20
2014	10	169	10
2016	10	132	10
2006	15	133	11
2015	10	218	10
2016	15	274	21
	19	271	21
2003	11	128	11
2009	15	143	13
1999	18	264	13
2011	15	128	19
2017	16	32	17
2014	9	148	9

For Review Only

Range = 1999 - 2017	Total = 1355	Median = 142	Total = 1344

For Review Only

Survival in Control group	Timing of Intervention	Efficacy	Quality Score
160	1	Y	5
100	1	Y	5
104	1	Y	5
168	1	Y	3
150	1	N	3
140	1	N	2
129	1	N	3
122	1	N	3
42	2	Y	3
125	1	Y	5
130	1	Y	2
165	3	Y	4
135	1	Y	4
124	3	Y	4
130	1	Y	4
134	1	Y	4
129	1	N	4
126	1	Y	4
119	1	Y	4
127	1	N	5
164	1	Y	6
124	1	Y	2
133	1	N	8
127	1	N	8
135	1	Y	6
115	2	Y	7
122	1	Y	4
297	1	Y	8
212	1	Y	4
152	1	Y	2
400	1	Y	4
132	1	Y	5
123	1	Y	4
146	2	Y	4
127	1	N	2
143	2	N	2
130	4	Y	4
128	1	N	4
140	1	Y	4
130	2	Y	4
7	1	Y	5
110	1	Y	5
101	2	Y	4

For Review Only

137	1	Y	5
122	1	Y	3
120	1	Y	6
123	1	N	6
140	1	N	6
141	2	N	4
127	1	Y	3
130	1	Y	5
124	1	Y	5
124	1	Y	6
140	1	Y	7
121	3	Y	3
124	1	Y	6
131	1	Y	4
124	1	Y	5
125	2	Y	5
127	1	Y	5
125	2	Y	5
140	1	Y	2
125	3	Y	5
123	1	Y	3
132	1	Y	5
141	1	N	5
163	1	N	5
120	1	Y	5
169	3	Y	8
162	1	Y	8
126	1	Y	4
126	1	Y	4
126	1	N	2
118	1	Y	5
134	1	Y	7
157	1	N	5
126	1	Y	5
163	1	N	6
143	2	N	3
126	1	Y	4
170	1	Y	3
254	2	Y	4
254	2	Y	4
137	1	N	5
132	1	Y	6
253	1	Y	4
125	1	N	6
23	1	Y	5
124	1	Y	4

For Review Only

Median = 129	Mode = 1	Y=68; N=21	Mode = 4

For Review Only

Putative pathway
MT
MT
MT; ETC
MT
MT; AI
AO; EX
AA
AA; MD; MT
AO
AA; AO
AA
AO; AA; AI
AO,AI, AA, MT, ETC
MT; ETC
AO; AI
AI
AA; MD
AI; AO
AO; AA
AO
AO; ETC
CB; MT
AO
MT
AO; AI; AA
MT; AO; AA; AI
CB; AO
AA; ETC; MT
AA; ETC
AI; MT
MT
CB; AA; AO
AO
EX
AI
MT; OT
OT; MD; AI
AO; AI

For Review Only

AA
EX
MT
AO
AO; MD; ETC
AO; AA
MT; AO
MD
MT
AA; AI
AA; AI; MD
AO; AI; MD
AO
MT
AO; MT
AA
AA; AO; EX
MT; MD
MD; AO; AI
AI; MT;
AI; MT
AO; AA
MD; AA
AO; MD; AA
AO; MD
MD
AA; AI
AI; MT
ETC; MT
AO
AI; AA; EX
MT; AO; AI
AO
AA
AO
AO AI
AA; AO
AA; AI

For Review Only

Mode = AO

For Review Only

Name of pathway
Preconditioned activated AMPK pathway
Delayed AMPK activation and consequent decreased ACC phosphorylation
ATP generation, boost ETC activity
Trophic factors
Fat metabolism; complement
CB2 receptor
mitochondrial integrity; GAPDH mediated apopototic pathway
Autophagy, mTOR, reduced morphological disruption of mitochondria
Anti-oxidant activity - not specified
upregulate endogenous neuronal apoptosis inhibitory protein leading to upregulation of anti-oxidativ
c-Abl inhibition
Reduced phosphorylation of p38 MAPK
CR increases lipid peroxidation, inflammation and apoptosis, while decreasing mitochondrial bioener
provide alternative energy source, boost ETC, increases basal and maximal mitochondrial oxygen con
Increased HSPs eg HSP70, reduces iNOS, TNF-a
inhibition of gliosis
"markers of apoptosis and mitochondrial dysfunction" - not specified
modifies NADPH oxidase 2 levels
Neu blocked the increase in reactive oxygen species (Mitotracker ROS) and lithium blocked Fas-media
increased mitochondrial DNA oxidation
inhibited the release of mitochondrial apoptogenic factors and the subsequent activation of downstr
sensitises the sarcomere to calcium
rho kinase inhibitor
Insulin-like growth factor
putatively: decreasing oxidative and free radical stress, stopping the inflammatory process, inhibiting
prevents weight loss and muscle catabolism
inhibits mitochondrial permeability transition pore; mitochondrial calcium retention
inhibitor of eukaryotic initiation factor 2α dephosphorylation, possesses anti-prion properties, attenu
bound directly to two components of the mitochondrial permeability transition pore: the voltage-dep
choroid plexus activation, and accumulation of immunoregulatory cells, and elevation of the neurotr
Trophic factors
protection of mitochondria through inhibition of the mitochondrial permeability transition
significant reduction in levels of malondialdehyde (a marker of lipid peroxidation), in total content of
P2X4 mediatiated pathway, potentiates effect of ATP on this receptor
reduced the expression of the proinflammatory cytokines Fas Ligand, IL-1beta, TNF-alpha and CD40 li
metabolic improvement, mitochondrial myopathy and also autophagy; higher mito oxidative capacity
activation of autophagy and an increase in the number of the mitochondria ie biogenesis in motor ne
better preservation of motor neuron architecture, less astrogliosis (glial fibrillary acidic protein), and

For Peer Review

inhibits the caspase-1/cytochrome c/caspase-3 cell death pathway, inhibits MT1 receptor loss
NMDA/ glutamate induced toxicity
switching to TCA cycle intermediates and arginine-alpha-ketoglutarate as fuel
anti-oxidant
NO synthetase inhibitor; free radical scavenger; increases autophagy
Caspase 1; caspase 3; iNOS inhibition; p38 mitogen-activated protein kinase inhibition; mitochondrial
Mitochondrial redox; pyruvate dehydrogenase complex stimulant
p62
Trophic factors
Cytochrome c; SMAC; AIF; BCL-XL; caspase 3; reduced IBA1 and GFAP
Apoptotic inhibitor; anti-inflammatory; reduced oxidative stress
Increased autophagy; reduced ROS; reduced microgliosis
NRF2
Mitochondrial creatine kinase increased; open mitochondrial transition pore
Slowed decline of mito function; reduced nitro oxidative markers
Cytochrome c; caspase 3; reduced abberant mitochondrial permeability
Apoptosis; p75; reduced ROS
Extracellular ATP receptor; mitophagy; global weight loss
Extracellular ATP receptor; mitophagy
proliferator-activated receptor-gamma agoinst
Complement
Reduced free radicals; decreased Bax, increased BCL-2
Increased mitophagy; decreased Bax and Caspase 3
Free radical scavenger; mitochondrial biogenesis/mitophagy; p53 (apoptosis) nd sirt1 downregulatio
Free radical scavenger; mitochondrial biogenesis/mitophagy
Upregulates mitophagy
BCL-2, NFkappaB, cytochrome c and caspase reduction
Extracellular ATP receptor; inflammatory cytokine reduction including NFkappaB
coenzyme Q10; heme A
ROS reduction
TNF-alpha; NFkappaB; increased glutamate transporter
MAP-Kinase regulating mitochondrial metabolism (JNK signalling); ROS reduction and; reduction in in
reduction in oxidative stress
Anti-apoptotic molecule (modified BCL-XL)
Free radical scavenger
Antioxidant; anti-inflammation
Apoptosis; oxidative strss reduction
Apoptosis; cytokines; VEGF; growth factors

For Review Only

For Review Only

Treatment	Target
PIP	CWD
PO	CWD
PO	CWD
CTS	CWD
PO	CWD
PIP	CWD
PO	MT
PO	CWD
PO	CWD
PIP	CWD
PO	CWD
PO	CWD
PO	CWD
PO	CWD
U	CWD
PIP	CWD
PO	CWD
PIP	CWD
PO	CWD
PO	CWD
PIP	CWD
PIV	CWD
PO	CWD
PSC	CWD
PSC	CWD
PSC	CWD
PIV	MT
PIP	CWD
PO; PIV	MT
PSC	CWD
CTS	CWD
PCNS	CWD
PIP	CWD
PO	CWD
PO	CWD
PO	CWD
PIP	CWD
PIP	CWD

For Review Only

PIP	CWD
PO	CWD
PO	CWD
PO	CWD
PO	CWD
PO	CWD
PO	MT
PO	CWD
CTU	CWD
PO	CWD
PO	CWD
PO	CWD
PO	CWD
PO	CWD
PO	MT
PO	CWD
PO	CWD
PO	CWD
PO	CWD
PO	CWD
PO	CWD
PO	CWD
PO	CWD
PO	CWD
PO	CWD
PO	CWD
PO	CWD
PO	CWD
PO	CWD
PO	MT
PIP	CWD
PO	MT
PO	CWD
CCNS	CWD
PSC	CWD
PO	CWD
GA	CWD
CTU	CWD

For Review Only

Mode = PO	Mode = CWD

For Review Only

Name of drug
latrepirdine
high-fat jelly diet
ketogenic diet, D-beta-3 hydroxybutyrate
Mesenchymal stem cells and olfactory enshething cells
Ganglioside
AM1241
CGP 3466B
n-butylidenephthalide
ginseng root (Panax quinquefolium)
bromocriptine
dasatinib
caffeic acid phenethyl ester
caloric restriction
Caprylic triglyceride
Celastrol
vincristine
Lithium
clemastine
2-hydroxy-5-(2,3,5,6-tetrafluoro-4-trifluoromethyl-benzylamino)-benzoic acid (Neu2000) AND lithiun
high polyunsaturated fatty acid content
Hydrogen-Rich Saline
Tirasemtiv
Fasudil
PEGylated insulin-like growth factor I
Gemals combinaton therapy, functional polypeptides (fatty acids, free radical scavengers and amino a
Ghrelin
GNX-4728
Guanabenz
cholest-4-en-3-one, oxime (TRO19622)
myelin-derived peptide
bone-marrow cell therapy
cyclosporin
iron porphyrin (FeTCPP)
ivermectin
Lenalidomide
Lipid-enriched diet
lithium
manganese porphyrin AEOL 10150 (manganese [III] tetrakis[N-N'-diethylimidazolium-2-yl]porphyrin)

melatonin
Memantine
Deanna protocol
Metformin
Methylene blue
Minocycline
Dichloroacetate
Trehlose
Human marrow stromal cells
N-acetyl-L-tryptophan
n-butylidenephthalide
bexarotene
CDDO ethylamide and CDDO trifluoroethylamide
Creatine
MitoQ
Nortriptyline
Clozapine
P2X7 antagonist (brilliant blue G)
P2X7 antagonist (brilliant blue G)
Pioglitazone
PMX205
VK-28 and M30
Rapamycin
Resveratrol
Resreratrol
Rilmenidine
Sodium phenylbutyrate
P2X7 antagonist (brilliant blue G)
Simvastatin
pramipexole derivative
peptide stearyl-norleucine-VIP
ASK-1 inhibitor
Zinc
Protein therapy
putrescine-modified catalase
VitD3
ASOs targeting ataxin2
Adipose tissue derived stem cells

For Review Only
