# Supplementary material for: Targeting mitochondrial dysfunction in amyotrophic lateral sclerosis: a systematic review and meta-analysis
Source: Brain Commun. 2019 Aug 6;1(1):fcz009. doi: 10.1093/braincomms/fcz009 (PMC7056361; doi:10.1093/braincomms/fcz009)
Supplement: fcz009_Supplementary_Data [file fcz009_supplementary_data.zip › Supplementary Files - Legends.docx]

**Supplementary Files – Legends** Mehta *et al.*

***Supplementary Table 1:***

Table summarising all data extracted from the 76 studies included in the quantitative meta-analysis.

***Supplementary Figure 1: Small sample size of non-SOD1 preclinical models reveals no statistically significant improvement in survival with therapies targeting mitochondrial dysfunction.***

**A.** Frequency distribution demonstrating the models implemented in the studies included in the meta-analysis. Data show that the *SOD1 G93A* mouse model was the most frequently used model. **B.** Forest plot including all non-SOD1 preclinical models: (i) TDP-43 models (*n* = 2), (ii) *pmn* model (*n* = 1), (iii) *wobbler* mouse model (*n* = 1), (iv) VCP mouse model (*n* = 1).
