# Supplementary figures and images for: Targeting mitochondrial dysfunction in amyotrophic lateral sclerosis: a systematic review and meta-analysis
Source: Brain Commun. 2019 Aug 6;1(1):fcz009. doi: 10.1093/braincomms/fcz009 (PMC7056361; doi:10.1093/braincomms/fcz009)

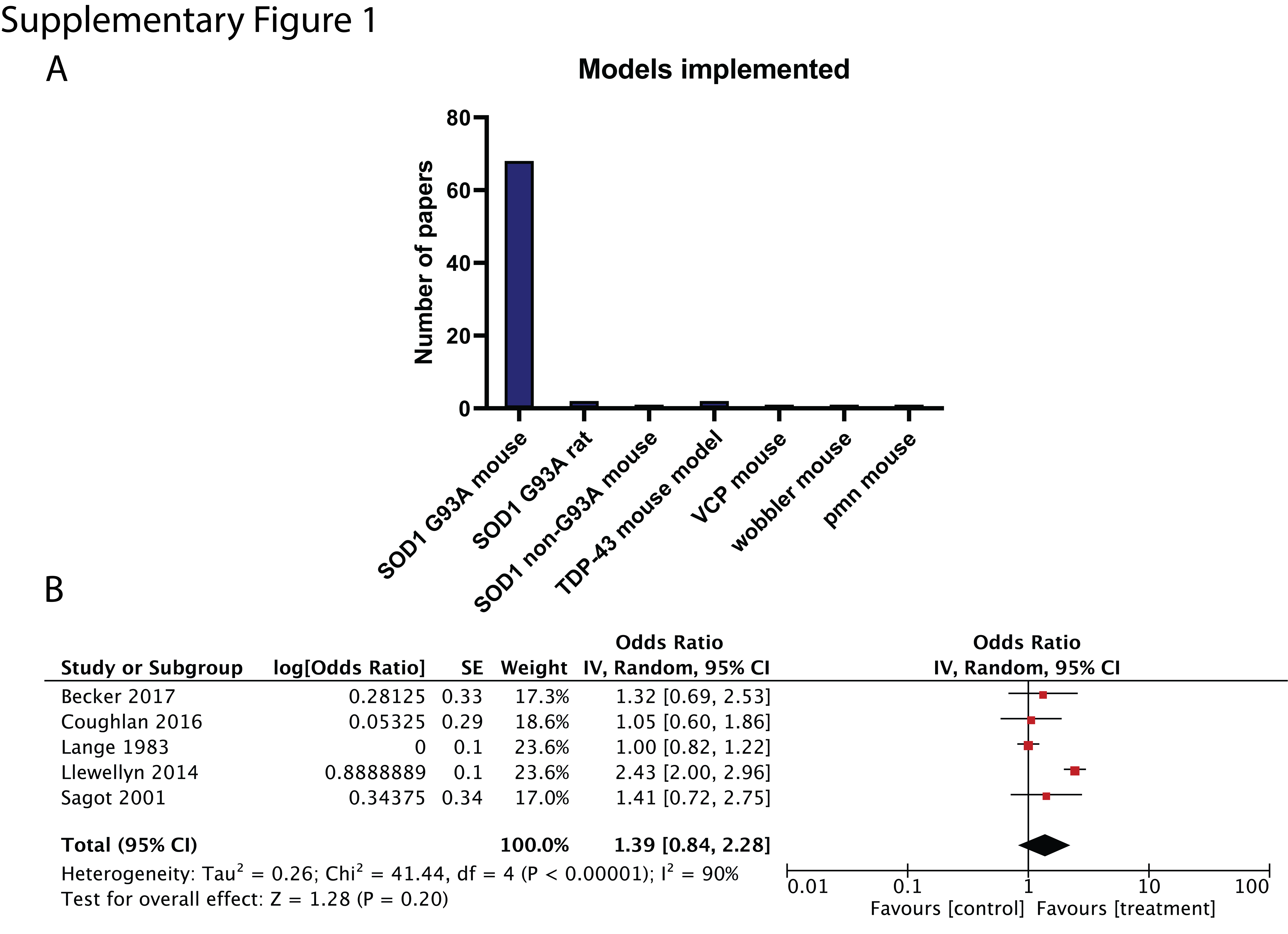

Supplement: fcz009_Supplementary_Data [file fcz009_supplementary_data.zip › Supplementary Figure 1.tif]
