# Supplementary material for: Targeting mitochondrial dysfunction in amyotrophic lateral sclerosis: a systematic review and meta-analysis
Source: Brain Commun. 2019 Aug 6;1(1):fcz009. doi: 10.1093/braincomms/fcz009 (PMC7056361; doi:10.1093/braincomms/fcz009)
Supplement: fcz009_Supplementary_Data [file BRAINCOM-2019-020_Response_to_Reviewers_Comments_Original_submission.pdf › BRAINCOM-2019-020_Response_to_Reviewers_Comments_Original_submission.pdf]

Reviewer: 1

Comments to the Author
The manuscript describes a meta-analysis of animal model data on interventions targeting mictochondria in ALS. It is an interesting topic which, to some extent, circumvents the question of whether mitochondrial dysfunction is important to the pathogenesis of ALS and specifically seeks evidence of whether intervening in mitochondrial physiology helps with the disease. The manuscript is well-written and the methodology pays heed to the established standards in the conduct of meta-analysis. Searches for eligible papers and screening is mostly clear, analysis is appropriate and data is presented logically. I have several issues which focus primarily on the choice of studies to include, with some appearing not to be directly related to mitochondrial physiology, and interpretation of the data presented vis a vis its relevance to human ALS due to reliance on the SOD1 G93A mouse model.

RESPONSE 1:
We agree that this point requires further clarification in both the Methods and in the Discussion, specifically to highlight the rationale and the limitations of such an approach. We have made the following changes to the text to address this point:

Methods:
"Noting that drugs can modulate multiple potential targets, and that the dogma of 'single drug, single target' is overly simplistic, here, for an intervention that targets mitochondria, we included any study stating an effect on this pathway, whether as a primary target or otherwise [see new references 1-3]. This is primarily to limit bias, with the aim of being comprehensive in our assessment of the literature. It is likely that different therapeutic interventions identified will target multiple, potentially non-overlapping, pathways. The intention is that all interventions identified will have mitochondrial pathways as a commonality and, therefore, through meta-analysis of many such interventions we will be able to identify a common therapeutic effect."

Discussion (under 'Study Limitations' section):
"First, the approach to identify potential therapeutic interventions was based on the identification of a statement by the authors in the papers identified that the intervention targeted putative mitochondrial pathways. This method was employed to limit bias and to identify as comprehensive a dataset as possible, cognisant that most drugs will have multiple target pathways, and it assumes that those identified will have mitochondrial targeting as a commonality. Notably, no approach to identifying such interventions is perfect, because of, sometimes unknown or not reported, off-target effects of drugs. If a study did not mention mitochondrial targeting in their study, we were not able to include it in our analysis. An example of this limitation would be studies examining the effects of rapamycin. Rapamycin is a drug known mostly for its effect on autophagy; however, rapamycin has also been shown to have multiple targets, including effects on mitochondrial dysfunction, primarily through its role in mitophagy, cellular metabolism and apoptosis [see new references 4-6]. Thus, as a result its divergent roles in different molecular pathways, and our inclusion criteria aimed at limiting bias, not all studies assessing the effect of rapamycin will have been included – only those that explicitly mentioned a role in mitochondrial pathways."

References:

1. Paolini GV, Shapland RHB, van Hoorn WP, Mason JS, Hopkins AL. Global mapping of pharmacological space. Nat Biotechnol. 2006;24:805–15.
2. Hopkins AL. Network pharmacology: the next paradigm in drug discovery. Nat Chem Biol. 2008;4:682–90.
3. Saha A, Varghese T, Liu A, Allen SJ, Mirzadegan T, Hack MD. An Analysis of Different Components of a High-Throughput Screening Library. J Chem Inf Model. 2018 Oct 22;58(10):2057-2068.
4. Li Q, Gao S, Kang Z, Zhang M, Zhao X, Zhai Y, Huang J, Yang GY, Sun W, Wang J. Rapamycin Enhances Mitophagy and Attenuates Apoptosis After Spinal Ischemia-Reperfusion Injury. Front Neurosci. 2018 Dec 3;12:865
5. Johnson SC, Martinez F, Bitto A, Gonzalez B, Tazaerslan C, Cohen C, Delaval L, Timsit J, Knebelmann B, Terzi F, Mahal T, Zhu Y, Morgan PG, Sedensky MM, Kaeberlein M, Legendre C, Suh Y, Canaud G. mTOR inhibitors may benefit kidney transplant recipients with mitochondrial diseases. Kidney Int. 2019 Feb;95(2):455-466.
6. Garza-Lombó C, Schroder A, Reyes-Reyes EM, Franco R. mTOR/AMPK signaling in the brain: Cell metabolism, proteostasis and survival. Curr Opin Toxicol. 2018 Apr;8:102-110.

Main points:
1. Although the methodology is mostly well-elucidated, it is not clear how the inclusion criterion "testing an intervention that targets mitochondria" was applied and the description needs expanding. In a number of the studies it is not clear how mitochondria are being specifically targeted, and many of the interventions have broad **systemic effects that are not primarily mediated by mitochondria** (e.g. those targeting the immune system, ataxin-2 etc.); although mitochondrial physiology might be altered by the interventions, in many of the studies this is not the primary aim and the improvement in survival cannot be confidently attributed to an effect on mitochondria.

RESPONSE 2:
We thank the reviewer for this comment; as detailed above, accepting that drugs have multiple targets - nothing can be confidently attributed to one pathway. We have employed a non-biased approach to identify papers with mitochondrial pathways as a commonality.

We would also like to highlight the supplementary data table, which specifically codes all pathways stated within the text of the included studies and the analysis of these pathways within Figure 4.

2. The meta-analysis is heavily reliant on the SOD1 G93A mouse model, which the authors acknowledge is an overexpression model that bears little relation to the vast majority of human ALS. The authors' comment "Thus, SOD1 transgenic models are likely to recapitulate only certain aspects of ALS." Could be levelled at any model and understates the problem. It would be helpful to include a discussion of why the SOD1 G93A mouse is relevant to mitochondrial mechanisms in ALS (does it recapitulate any of the findings in human tissue discussed in the introduction, for example, or other models?). I would therefore suggest subgroup analysis to examine non-SOD1-based models separately, given the skepticism as to their relevance to most cases of ALS.

RESPONSE 3:
We thank the reviewer for this suggestion and have added these data as a subgroup analysis in the supplementary data (Supplementary Figure 1) and have added the following additional text:

Thus, SOD1 transgenic models are likely to recapitulate only certain aspects of ALS; notwithstanding this, there is emerging evidence that misfolded SOD1 protein is pathologically present in sporadic ALS with no known genetic cause (new ref 7), and in cases of ALS caused by mutations in known ALS genes other than SOD1 (new ref 8), suggesting that SOD1 biology may well be involved in ALS pathogenesis in general. With respect to mitochondrial neurobiology, aberrations in mitochondrial morphology, function, clearance and transport have been observed in mutant SOD1 mice (new ref 9).

References:

7. Paré B, Lehmann M, Beaudin M, Nordström U, Saikali S, Julien JP, et al. Misfolded SOD1 pathology in sporadic Amyotrophic Lateral Sclerosis. Sci Rep 2018; 8(1): 14223.
8. Forsberg K, Graffmo K, Pakkenberg B, Weber M, Nielsen M, Marklund S, et al. Misfolded SOD1 inclusions in patients with mutations in. J Neurol Neurosurg Psychiatry 2019.
9. Shi P, Gal J, Kwinter DM, Liu X, Zhu H. Mitochondrial dysfunction in amyotrophic lateral sclerosis. Biochim Biophys Acta 2010; 1802(1): 45-51.

Minor points:
Abstract:
3. "The extant preclinical literature indicates that targeting mitochondrial dysfunction **prolongs survival** in ALS, particularly if the intervention is administered early" needs qualification: the authors highlight many substandard aspects of the included studies including the overreliance on SOD1 models which suggest that such a generalization is excessive.

RESPONSE 4:
We have now changed the text to "may prolong survival."

Introduction:
4. The introduction gives a nice succinct discussion of some evidence for mitochondrial mechanisms in ALS but does not deal with counterarguments (e.g. lack of genes) or the breadth of other implicated pathways; it also does not deal with the lack of translation of mitochondrial interventions, notwithstanding edaravone, or that mitochondrial dysfunction is implicated in other neurodegenerative diseases.

RESPONSE 5:
We have added the following text to the Introduction to address this: "Finally, not only are there a plethora of ALS-causing mutations that have been identified that impact on mitochondrial function (new ref 10), there is also direct evidence that disruption of mitochondrial structure and function contributes to the aetiology of ALS, owing to the discovery of causative mutations in the gene encoding the mitochondrial protein, CHCHD10, which is localised to contact sites between the inner and outer mitochondrial membrane (new ref 11)."

10. Smith EF, Shaw PJ, De Vos KJ. The role of mitochondria in amyotrophic lateral sclerosis. Neurosci Lett 2017.
11. Bannwarth S, Ait-El-Mkadem S, Chaussenot A, Genin EC, Lacas-Gervais S, Fragaki K, et al. A mitochondrial origin for frontotemporal dementia and amyotrophic lateral sclerosis through CHCHD10 involvement. Brain 2014; 137(Pt 8): 2329-45.

Results:
5. It would be helpful to see more estimates of effect size (i.e. confidence intervals)

RESPONSE 6:
We have provided effect size estimates and confidence intervals for all of the meta-analyses included.

6. A summary of the models would be helpful (e.g. what number were SOD1 mouse, non-SOD1 mouse, rat, c elegans, zebrafish etc.)

RESPONSE 7:

We thank the reviewer for this suggestion and we agree that these data will provide clarity and we have included a frequency distribution in Supplementary Figure 1A.

Discussion:
5. In relation to point 2, many of the interventions are likely to be promiscuous in their effects i.e. affect multiple pathways not restricted to mitochondrial physiology and this should at least be acknowledged.

RESPONSE 8:
This is acknowledged in Figure 4 and the Supplementary Material and now referred to in the text.

6. "The most targeted pathway in the extant preclinical literature is the antioxidative stress pathway" of all pathways? Or just the mitochondrial pathways?

RESPONSE 9:
We agree with the need for clarification on this. This is now acknowledged in Figure 4 and the Supplementary Material, and now referred to in the text as per the Discussion (and additional text) indicated above (in response 1).

7. No mention is made of the difficulties or means to translate treatments that are most effective in the presymptomatic phase.

RESPONSE 10:
See Figure 5 and related text and additional text in the Discussion to address this point:
"There is clearly a focus in the current literature on pre-symptomatic treatment of animals, which makes these therapies more difficult to translate to the clinic. Whilst genetic testing could identify individuals with potential for pre-symptomatic intervention, the majority of ALS cases are not caused by a known genetic mutation. Furthermore, there is no existing biomarker for the detection of early disease states in these cases. We therefore recommend that preclinical studies focus on interventions at or after symptom onset to improve the translational potential of these studies."

Reviewer: 2

Comments to the Author
In this manuscript the authors perform a meta-analysis of preclinical research in ALS with respect to mitochondria-targeting interventions. Their main conclusion is that targeting mitochondrial dysfunction improves survival in preclinical models when administered prior to onset.

To a non-expert the methodology appears appropriate.

I am concerned about the rather sweeping conclusions of this review.
Scanning the titles of the include manuscripts, most do not appear to treat with mitochondrial drugs so I don't believe it is accurate it is to say that targetting mitochondria improves survival. For example the authophagy enhancers like trehalose, rilminedine and rapamycin are more likely to act on protein aggregates.

RESPONSE 11:
See comment above regarding other pathways involved. This has now been addressed as stated in the comments for Reviewer 1 (response 1).

In this respect i note that another study on rapamycin (with the oposite outcome as the one in this study) was not included - is this because it did not mention mitochondria?

RESPONSE 12:
This would not have been included due to the lack of reference to mitochondria (our inclusion criteria; see comments above in response 1). This has been specifically addressed in the revised discussion as an example.

Along the same lines - how does lowevering ataxin in a TDP-43 model target mitochondria, or inhibition of complement signaling? And I could go on.

RESPONSE 13:
Ataxin 3, as well as its well-established role in proteostasis, has also been shown to localize to mitochondria and effect apoptotic pathways as well as mitochondrial morphology, function and homeostasis.

- Pozzi C, Valtorta M, Tedeschi G, Galbusera E, Pastori V, Bigi A, Nonnis S, Grassi E, Fusi P. Study of subcellular localization and proteolysis of ataxin-3. Neurobiol Dis. 2008 May;30(2):190-200.
- Harmuth T, Prell-Schicker C, Weber JJ, Gellerich F, Funke C, Drießen S, Magg JCD, Krebiehl G, Wolburg H, Hayer SN, Hauser S, Krüger R, Schöls L, Riess O, Hübener-Schmid J. Mitochondrial Morphology, Function and Homeostasis Are Impaired by Expression of an N-terminal Calpain Cleavage Fragment of Ataxin-3. Front Mol Neurosci. 2018 Oct 10;11:368.

- Kristensen LV, Oppermann FS, Rauen MJ, Fog K, Schmidt T, Schmidt J, Harmuth T, Hartmann-Petersen R, Thirstrup K. Mass spectrometry analyses of normal and polyglutamine expanded ataxin-3 reveal novel interaction partners involved in mitochondrial function. Neurochem Int. 2018 Jan;112:5-17.
- Chou AH, Lin AC, Hong KY, Hu SH, Chen YL, Chen JY, Wang HL. p53 activation mediates polyglutamine-expanded ataxin-3 upregulation of Bax expression in cerebellar and pontine nuclei neurons. Neurochem Int. 2011 Feb;58(2):145-52
- Tsai HF, Tsai HJ, Hsieh M. Full-length expanded ataxin-3 enhances mitochondrial-mediated cell death and decreases Bcl-2 expression in human neuroblastoma cells. Biochem Biophys Res Commun. 2004 Nov 26;324(4):1274-82.

Not unexpected, most studies involve the SOD1 model I don't think that from this dataset the authors can claim that targetting mitochondria (if at al - see above) is effective for all ALS; this appears to be a SOD1 specific phenomenon. Is it possible to compare the SOD1 model with the other models included in the Review - is the same effect observed?

RESPONSE 14:
We have undertaken this suggested subgroup analysis and included it as a supplementary figure (see comment above and supplemental figure 1). This post hoc subgroup analysis revealed that, whilst the effect size did favour therapies targeting mitochondrial pathways, the result did not reach statistical significance and that there are likely too few studies to come to a definitive conclusion at this point.

Other Comments:
I was suprised to see "yeast" included as animal model - one could argue that as a single cell organism it may be more similar to cell culture models? The authors state all the studies retained in the Review where using rodent models- can they comment why the other models where excluded? Is this because they typically don't look at survival?

RESPONSE 15:
“Yeast” was included in our search terms as it is a preclinical model. However, no studies were identified testing therapies on yeast models. No models were excluded. Studies were only excluded if they did not meet our inclusion criteria.

The authors note that most studies show serious flaws in methodology - how is this addressed in the analysis, should these studies be excluded? How do the "flawed" studies compare to the others - is the same effect observed?

RESPONSE 16:
Figure 5C and the associated text assess whether the quality of the study had any impact on the likelihood of demonstrating efficacy in the study. We demonstrate that the quality of studies did not impact upon the likelihood of demonstrating efficacy. We also performed a recalculation of the overall effect size owing to an outlier study that resulted in an over-estimate of the original effect size.

The question regarding whether poor quality studies should be excluded is very interesting. We agree with the reviewer that this is an important methodological decision. Our quality assessment was intended as a structured assessment of the literature. It is difficult to use a single quality checklist to exclude studies as checklist items are not considered to be equal per se. For example, a “power calculation” and a “statement of temperature control” could have very different effects on the quality of the study. Also, items such as “statement regarding randomization” may not be relevant for the study. Furthermore, the authors of a study may have controlled temperature, but just not stated it in their methods. Moreover, reporting of quality checklist items (especially power calculations) may have improved over time, making it difficult to compare between studies, conducted over different time periods. We therefore chose to implement the quality checklist to assess the quality of the literature in a structured, non-biased manner to allow us to comment on areas for future improvement rather than to exclude studies, which may lead to unconscious bias.

Along the same lines, the authors discuss other limitations to the Review such as differences in high and low copy number SOD1 mice; Could some of these limitations be addressed by analysing and comparing subgroups?

RESPONSE 17:
We reanalyzed our data and identified that 21 studies stated that they used the high copy number mouse model; 3 studies stated that they used the low copy number mouse model and 57 (65% of the included studies) did not state the model used. We have now stated this in the text and included this information in the supplementary data table.
