# Supplementary material for: Targeting mitochondrial dysfunction in amyotrophic lateral sclerosis: a systematic review and meta-analysis
Source: Brain Commun. 2019 Aug 6;1(1):fcz009. doi: 10.1093/braincomms/fcz009 (PMC7056361; doi:10.1093/braincomms/fcz009)
Supplement: fcz009_Supplementary_Data [file BRAINCOM-2019-020_Response_to_Reviewers_Comments_Revision_1.pdf › BRAINCOM-2019-020_Response_to_Reviewers_Comments_Revision_1.pdf]

12th July 2019

RE: Revised manuscript submission for "**Targeting mitochondrial dysfunction in amyotrophic lateral sclerosis – a systematic review and meta-analysis of preclinical research (BRAINCOM-2019-020.R1**)" for publication in *Brain Communications*.

Dear Professor Talbot,

We thank you for the opportunity to re-submit our revised manuscript in response to the editorial and reviewer's comments. Specifically, we have included the study limitations caveat within our abstract, as you suggested. Furthermore, we have included an abbreviated summary and graphical abstract, as requested.

We now believe this manuscript addresses the concerns of the reviewers and our full response to each of the Reviewer's comments is detailed point-by-point below at the foot of this letter.

Yours sincerely,

Dr Jenna Gregory

Reviewer(s)' Comments to Author:

Reviewer: 1

Comments to the Author
The authors have attempted to address my concerns about the first version of the manuscript. I am still very concerned that the model contributing most of the data is of dubious relevance to our understanding of TDP-43 ALS. The reliance on the SOD1 G93A mouse and the lack of significant effect in non-SOD1 models ought to be emphasised in the abstract.

We have included this suggested concession in the conclusions of our abstract for clarity:
"The extant preclinical literature indicates that targeting mitochondrial dysfunction may prolong survival in ALS, particularly if the intervention is administered early. A limitation of current research is a significant bias towards models based on SOD1, with uncertainty about generalisability to ALS due to TDP-43 proteinopathy. However, further mechanistic research is clearly warranted in this field."
There is also what I assume to be a typographic error in the discussion "out of the 76 studies that were included in the meta-analysis, 81 used SOD1 G93A
transgenic mouse models".

This is indeed a typographical error and has been corrected in the revised manuscript.
