# Supplementary material for: Targeting mitochondrial dysfunction in amyotrophic lateral sclerosis: a systematic review and meta-analysis
Source: Brain Commun. 2019 Aug 6;1(1):fcz009. doi: 10.1093/braincomms/fcz009 (PMC7056361; doi:10.1093/braincomms/fcz009)
Supplement: fcz009_Supplementary_Data [file BRAINCOM-2019-020_Decision_Original_submission.pdf › BRAINCOM-2019-020_Decision_Original_submission.pdf]

23-May-2019

Dear Dr. Gregory,

Thank you for your recent submission BRAINCOM-2019-020 entitled "Targeting mitochondrial dysfunction in amyotrophic lateral sclerosis – a systematic review and meta-analysis of preclinical research" to Brain Communications.

Your manuscript has been reviewed, and while the reviewers and editorial team appreciate many aspects of the paper, they raise some concerns that you will need to address in order for the paper to be considered for publication.

Therefore, I invite you to respond to the reviewer(s)' comments and revise your manuscript. The reviewer(s)' comments are included at the bottom of this message.

In addition to the reviewers' comments, please note our manuscript formatting guidelines which can be found here:

https://academic.oup.com/braincomms/pages/General_Instructions

In particular, please note in bar graphs where n<10, each data point should be shown on the graph.

To revise your manuscript, click the link below or log into https://mc.manuscriptcentral.com/braincom and enter your Author Centre, where you will find your manuscript title listed under "Manuscripts with Decisions." Under "Actions," click on "Create a Revision." Your manuscript number has been appended to denote a revision.

*** PLEASE NOTE: This is a two-step process. After clicking on the link, you will be directed to a webpage to confirm. ***

https://mc.manuscriptcentral.com/braincom?URL_MASK=d6ff6d1b0bbc48f982c1f8273a5c155c

Please upload a revised manuscript with changes tracked with track changes or in coloured text.

When submitting your revised manuscript, you will be asked to respond to the reviewer(s) comments. Please be as specific as possible in your response to all of the comments.

The editorial team at Brain Communications aims to increase transparency in scientific publishing and to demonstrate the value that peer review brings to the scientific process. Please therefore consider whether you are willing to have reviewer(s)' comments on your paper and your response published as supplementary data with the paper if it is accepted. This is entirely optional and will only be done if both the authors and reviewers agree. While submitting your revision there will be an opportunity to review or update the answer you gave previously, if necessary.

IMPORTANT: Your original files are available to you when you upload your revised manuscript. Please delete any redundant files before completing the submission.

Because we are trying to facilitate timely publication of manuscripts submitted to Brain Communications, we would appreciate your revised manuscript as soon as possible. If it is not possible for you to resubmit within 3 months, the option to submit a revision will expire, and we would then consider your revised paper as a new submission. However, if you need a little more time, please get in touch and we can extend this deadline for you.

Once again, thank you for submitting your manuscript to Brain Communications and I look forward to receiving your revision.

Yours sincerely,
Prof. Kevin Talbot
Editor, Brain Communications

Reviewer(s)' Comments to Author:

Reviewer: 1

Comments to the Author
The manuscript describes a meta-analysis of animal model data on interventions targeting mictochondria in ALS. It is an interesting topic which, to some extent, circumvents the question of whether mitochondrial dysfunction is important to the pathogenesis of ALS and specifically seeks evidence of whether intervening in mitochondrial physiology helps with the disease. The manuscript is well-written and the methodology pays heed to the established standards in the conduct of meta-analysis. Searches for eligible papers and screening is mostly clear, analysis is appropriate and data is presented logically. I have several issues which focus primarily on the choice of studies to include, with some appearing not to be directly related to mitochondrial physiology, and interpretation of the data presented vis a vis its relevance to human ALS due to reliance on the SOD1 G93A mouse model.

Main points:
1. Although the methodology is mostly well-elucidated, it is not clear how the inclusion criterion "testing an intervention that targets mitochondria" was applied and the description needs expanding. In a number of the studies it is not clear how mitochondria are being specifically targeted, and many of the interventions have broad systemic effects that are not primarily mediated by mitochondria (e.g. those targeting the immune system, ataxin-2 etc.); although mitochondrial physiology might be altered by the interventions, in many of the studies this is not the primary aim and the improvement in survival cannot be confidently attributed to an effect on mitochondria.

2. The meta-analysis is heavily reliant on the SOD1 G93A mouse model, which the authors acknowledge is an overexpression model that bears little relation to the vast majority of human ALS. The authors' comment "Thus, SOD1 transgenic models are likely to recapitulate only certain aspects of ALS." Could be levelled at any model and understates the problem. It would be helpful to include a discussion of why the SOD1 G93A mouse is relevant to mitochondrial mechanisms in ALS (does it recapitulate any of the findings in human tissue discussed in the introduction, for example, or other models?). I would therefore suggest subgroup analysis to examine non-SOD1-based models separately, given the skepticism as to their relevance to most cases of ALS.

Minor points:
Abstract:
3. "The extant preclinical literature indicates that targeting mitochondrial dysfunction prolongs survival in ALS, particularly if the intervention is administered early" needs qualification: the authors highlight many substandard aspects of the included studies including the overreliance on SOD1 models which suggest that such a generalization is excessive.

Introduction:
4. The introduction gives a nice succinct discussion of some evidence for mitochondrial mechanisms in ALS but does not deal with counterarguments (e.g. lack of genes) or the breadth of other implicated pathways; it also does not deal with the lack of translation of mitochondrial interventions, notwithstanding edaravone, or that mitochondrial dysfunction is implicated in other neurodegenerative diseases.

Results:
5. It would be helpful to see more estimates of effect size (i.e. confidence intervals)

6. A summary of the models would be helpful (e.g. what number were SOD1 mouse, non-SOD1 mouse, rat, c elegans, zebrafish etc.)

Discussion:
5. In relation to point 2, many of the interventions are likely to be promiscuous in their effects i.e. affect multiple pathways not restricted to mitochondrial physiology and this should at least be acknowledged.

6. "The most targeted pathway in the extant preclinical literature is the antioxidative stress pathway" of all pathways? Or just the mitochondrial pathways?

7. No mention is made of the difficulties or means to translate treatments that are most effective in the presymptomatic phase.

Reviewer: 2

Comments to the Author
In this manuscript the authors perform a meta-analysis of preclinical research in ALS with respect to mitochondria-targeting interventions. Their main conclusion is that targeting mitochondrial dysfunction improves survival in preclinical models when administered prior to onset.

To a non-expert the methodology appears appropriate.

I am concerned about the rather sweeping conclusions of this review.
Scanning the titles of the include manuscripts, most do not appear to treat with mitochondrial drugs so I don't believe it is accurate it is to say that targetting mitochondria improves survival. For example the authophagy enhancers like trehalose, rilminedine and rapamycin are more likely to act on protein aggregates. In this respect i note that another study on rapamycin (with the oposite outcome as the one in this study) was not included - is this because it did not mention mitochondria? Along the same lines - how does lowevering ataxin in a TDP-43 model target mitochondria, or inhibition of complement signaling? And I could go on. Thus it appears that the conclusion that targeting mitochondria improves survival may be somewhat misleading?
Not unexpected, most studies involve the SOD1 model I don't think that from this dataset the authors can claim that targetting mitochondria (if at al - see above) is effective for all ALS; this appears to be a SOD1 specific phenomenon. Is it possible to compare the SOD1 model with the other models included in the Review - is the same effect observed?

Other Comments:
I was suprised to see "yeast" included as animal model - one could argue that as a single cell organism it may be more similar to cell culture models? The authors state all the studies retained in the Review where using rodent models- can they comment why the other models where excluded? Is this because they typically don't look at survival?

The authors note that most studies show serious flaws in methodology - how is this addressed in the analysis, should these studies be excluded? How do the "flawed" studies compare to the others - is the same effect observed?

Along the same lines, the authors discuss other limitations to the Review such as differences in high and low copy number SOD1 mice; Could some of these limitations be addressed by analysing and comparing subgroups?
