# Supplementary material for: Targeting mitochondrial dysfunction in amyotrophic lateral sclerosis: a systematic review and meta-analysis
Source: Brain Commun. 2019 Aug 6;1(1):fcz009. doi: 10.1093/braincomms/fcz009 (PMC7056361; doi:10.1093/braincomms/fcz009)
Supplement: fcz009_Supplementary_Data [file BRAINCOM-2019-020_Decision_revision_1.pdf › BRAINCOM-2019-020_Decision_revision_1.pdf]

10-Jul-2019

Dear Dr. Gregory,

Thank you for your recent submission BRAINCOM-2019-020.R1
entitled "Targeting mitochondrial dysfunction in amyotrophic lateral sclerosis – a systematic review and meta-analysis of preclinical research" to Brain Communications.

Your revised manuscript has been re-reviewed. The reviewer(s)' comments are included at the bottom of this message. Both reviwers have highlighted the limitations of the study, given that the bulk of the analysis relies on data from SOD1 models. While they agree that you have made this explicit in the main text, they feel, and I agree, that it would be important to make this point clear in the absract. Since this is a metanalysis, you could add an extra field to teh abstract for 'limitations of this study', with 1-2 sentences making the point. Alternatively, you could rephrase the conclusion section to read:

"The extant preclinical literature indicates that targeting mitochondrial dysfunction may prolong survival in ALS, particularly if the intervention is administered early. A limitation of current research is a significant bias towards models based on SOD1, with uncertainty about generalisability to ALS due to TDP-43 proteinopathy. However, further mechanistic research is clearly warranted in this field.

In addition to the reviewers' comments, please note our manuscript formatting guidelines which can be found here:

https://academic.oup.com/braincomms/pages/General_Instructions

Please note that for the final accepted paper, we will require an abbreviated summary of no more than 50 words and a graphical abstract.

To revise your manuscript, click the link below or log into https://mc.manuscriptcentral.com/braincom and enter your Author Centre, where you will find your manuscript title listed under "Manuscripts with Decisions." Under "Actions," click on "Create a Revision." Your manuscript number has been appended to denote a revision.

*** PLEASE NOTE: This is a two-step process. After clicking on the link, you will be directed to a webpage to confirm. ***

https://mc.manuscriptcentral.com/braincom?URL_MASK=3baa7f1005ee43e5bd1538ec1de63df9

Please upload a revised manuscript with changes tracked using track changes or in coloured text.

When submitting your revised manuscript, you will be asked to respond to the reviewer(s) comments. Please be as specific as possible in your response to all of the comments.

The editorial team at Brain Communications also aims to increase transparency in scientific publishing and to demonstrate the value that peer review brings to the scientific process. Please therefore consider whether you are willing to have reviewer comments on your paper and your response published as supplementary data with the paper if it is accepted. This is entirely optional and will only be done if both the authors and reviewers agree. When submitting your revision you will have the opportunity to update the answer you entered previously.

IMPORTANT: Your original files are available to you when you upload your revised manuscript. Please delete any redundant files before completing the submission.

Because we are trying to facilitate timely publication of manuscripts submitted to Brain Communications, we would appreciate your revised manuscript as soon as possible. If it is not possible for you to resubmit within 3 months, the option to submit a revision will expire, and we would then consider your revised paper as a new submission. However, if you need a little more time, please get in touch and we can extend this deadline for you.

Once again, thank you for submitting your manuscript to Brain Communications and I look forward to

receiving your revision.

Yours sincerely,
Prof. Kevin Talbot
Editor, Brain Communications

Reviewer(s)' Comments to Author:

Reviewer: 1

Comments to the Author
The authors have attempted to address my concerns about the first version of the manuscript. I am still very concerned that the model contributing most of the data is of dubious relevance to our understanding of TDP-43 ALS. The reliance on the SOD1 G93A mouse and the lack of significant effect in non-SOD1 models ought to be emphasised in the abstract.

There is also what I assume to be a typographic error in the discussion "out of the 76 studies that were included in the meta-analysis, 81 used SOD1 G93A
transgenic mouse models".

Reviewer: 2

Comments to the Author
