# Supplementary material for: Targeting mitochondrial dysfunction in amyotrophic lateral sclerosis: a systematic review and meta-analysis
Source: Brain Commun. 2019 Aug 6;1(1):fcz009. doi: 10.1093/braincomms/fcz009 (PMC7056361; doi:10.1093/braincomms/fcz009)
Supplement: fcz009_Supplementary_Data [file BRAINCOM-2019-020_Decision_revision_2.pdf › BRAINCOM-2019-020_Decision_revision_2.pdf]

23-Jul-2019

Dear Dr. Gregory,

It is a pleasure to accept your manuscript entitled "Targeting mitochondrial dysfunction in amyotrophic lateral sclerosis – a systematic review and meta-analysis of preclinical research" for publication in Brain Communications.

The next step is for your paper to go through "First Look". We will contact you again shortly if any final files or formatting changes are required for publication or if we have any queries.

If no changes are needed, your submission will be transferred to our Production Department and you will hear from the Production Editor in due course.

Thank you very much for your contribution. On behalf of the Editors of Brain Communications, we look forward to your future contributions to the Journal.

Yours sincerely,

Professor Kevin Talbot
Associate Editor
Brain Communications
